# *Porphyromonas gingivalis* Promotes Neuroinflammation by Microglial Ferroptosis via NOX4/PPAR-α/PGC-1α Pathway

**DOI:** 10.34133/research.1163

**Published:** 2026-04-08

**Authors:** Xue Li, Zhenhuan Wu, Chao Yao, Pengye Zhang, Qingyu Zhu, Shunxue Nan, Lei Xiao, Shengcai Qi

**Affiliations:** ^1^ Department of Prosthodontics, Shanghai Stomatological Hospital, Fudan University, Shanghai 200001, China.; ^2^ Shanghai Key Laboratory of Craniomaxillofacial Development and Diseases, Fudan University, Shanghai 200002, China.; ^3^ School of Stomatology, Fudan University, Shanghai 200032, China.; ^4^ The State Key Laboratory of Medical Neurobiology, MOE Frontiers Centre for Brain Science, and the Institutes of Brain Science, Fudan University, Shanghai 200032, China.

## Abstract

**Background:** Emerging evidence links *Porphyromonas gingivalis* (*P.g*), a keystone oral pathogen, neuroinflammation as a driver of Alzheimer’s symptoms. This study aimed to investigate the molecular mechanism by which *P.g* triggers neuroinflammation and cognitive decline. **Methods:** Wild-type (WT) mice were orally gavaged with *P.g* for 8 weeks to evaluate cognitive function, neuronal integrity (p-Tau, hippocampal damage), and neuroinflammation. RNA sequencing analyzed brain transcriptomic, ferroptosis, and mitochondrial function after *P.g* induction was mainly analyzed. In vitro, the roles of NOX4/PPAR-α/PGC-1α pathway on ferroptosis, mitochondrial function, and inflammatory responses were evaluated after microglia were treated with *P.g* supernatant. Finally, *NOX4*-knockout mice were used to validate pathway specificity. **Results:** *P.g* administration in WT mice induced cognitive deficits, hippocampal neurodegeneration, p-Tau accumulation, and neuroinflammation, accompanied by dysregulated mitochondrial genes (NOX4, PPAR-α, and PGC-1α) and ferroptosis activation. *P.g* supernatant promoted microglial ferroptosis, mitochondrial dysfunction, and inflammatory cytokine release in vitro, which were reversed by NOX4 silencing. Mechanistically, *NOX4* knockdown restored PPAR-α/PGC-1α signaling, suppressed ferroptosis, and mitigated inflammation in vitro. Critically, *NOX4*-knockout mice resisted *P.g*-induced cognitive impairment, neuronal loss, and neuroinflammatory responses in vivo. **Conclusion:** This study identified *P.g*-induced neuroinflammation and cognitive decline via microglial ferroptosis and mitochondrial dysfunction, which were regulated by the NOX4/PPAR-α/PGC-1α pathway. These findings highlight the link between oral health and brain pathology in Alzheimer’s disease and propose NOX4 as a promising pharmacological target for cognitive preservation.

## Introduction

Among neurodegenerative disorders, Alzheimer’s disease (AD) stands as the foremost contributor to dementia cases worldwide [1,2]. The number of AD patients will reach 152 million in 2050 [3]. However, as the underlying pathology of AD is not fully elucidated, effective treatment strategies are still lacking [4]. Epidemiological studies indicate that patients with chronic periodontitis (CP) have a higher risk of developing AD [5]. The association between periodontitis and AD is well-documented. However, the underlying mechanisms by which CP contributes to AD remain elusive.

CP is a chronic oral bacterial infection, suggesting that periodontal pathogens may be implicated in the onset and progression of AD [6]. *Porphyromonas gingivalis* (*P.g*), a highly virulent gram-negative bacterium, plays a key role in linking CP to systemic inflammatory conditions [6]. A study observed a more than 6-fold increase in the occurrence of AD once *P.g* was detected in the brain [7]. Evidence from recent studies indicates that *P.g* infection is capable of eliciting central pathological features of AD in mice, such as neuroinflammation and amyloid-β accumulation [8,9]. However, the precise mechanisms of *P.g* on the progression of AD need further investigation.

Ilievski et al. [10] demonstrated that repeatedly administering *P.g* orally to wild-type (WT) mice resulted in its translocation to the brain. Researchers detected the bacterium and its gingipains in microglia, astrocytes, neurons, and the extracellular space. Microglia, the central nervous system’s (CNS) tissue-resident macrophages, represent 5% to 10% of all cells and function as its primary line of immune defense [10]. The release of inflammatory cytokines from chronically activated microglia promotes neurodegeneration and contributes to the progression of AD [11,12]. Furthermore, *P.g* lipopolysaccharide (LPS) has been detected in the human brain and also has been proven to activate microglia in the brain [9,13]. Therefore, exploring the pathogenic mechanism of *P.g* affecting microglia in AD progression is important.

Mitochondrial damage is known to trigger excessive reactive oxygen species (ROS) production and disrupt iron homeostasis, contributing to the pathological basis of neurodegeneration like AD and periodontitis [14]. Microglial mitochondrial metabolism is essential for regulating brain bioenergetics, neurotransmission, and redox balance [15,16]. During oxidative stress, mitochondrial metabolism primarily generates ROS, which mediate intracellular signaling [17,18]. Extensive evidence demonstrates that the generation of ROS during ferroptosis is primarily driven by the Fenton reaction [19]. Moreover, NADPH-dependent ROS generation critically contributes to ferroptosis, particularly in the CNS [20]. NADPH oxidase 4 (NOX4), peroxisome proliferator-activated receptor α (PPAR-α), and peroxisome proliferator-activated receptor γ coactivator 1α (PGC-1α) form a core regulatory axis governing mitochondrial function and cellular metabolism. As a primary mitochondrial ROS (mtROS) source, NOX4 orchestrates mitochondrial dynamics and biogenesis via PPAR-α/PGC-1α signaling—a cascade essential for metabolic stress adaptation. This pathway critically influences cardiovascular integrity, cancer progression, and neurodegeneration through mitochondrial turnover, bioenergetics, and antioxidant regulation [21,22]. PGC-1α governs the expression of mitochondrial components, establishing it as both a key regulator of mitochondrial biogenesis and a key modulator of cellular antioxidant defense [23]. These studies indicate that mitochondria damage caused by NOX4, PPAR-α, and PGC-1α may be involved in *P.g* affecting microglia in AD progression.

This study focused on the mechanistic role of *P.g* in triggering microglial neuroinflammation during AD. A mouse periodontitis model was established through chronic oral administration of *P.g*. Subsequently, we assessed cognitive function, neuronal activity, and neuroinflammatory responses, with a specific focus on microglial ferroptosis and mitochondrial function. The underlying molecular mechanisms were further analyzed*.*

## Results

### *P.g* significantly aggravates anxiety and cognitive impairment in mice

**Fig. 1.**
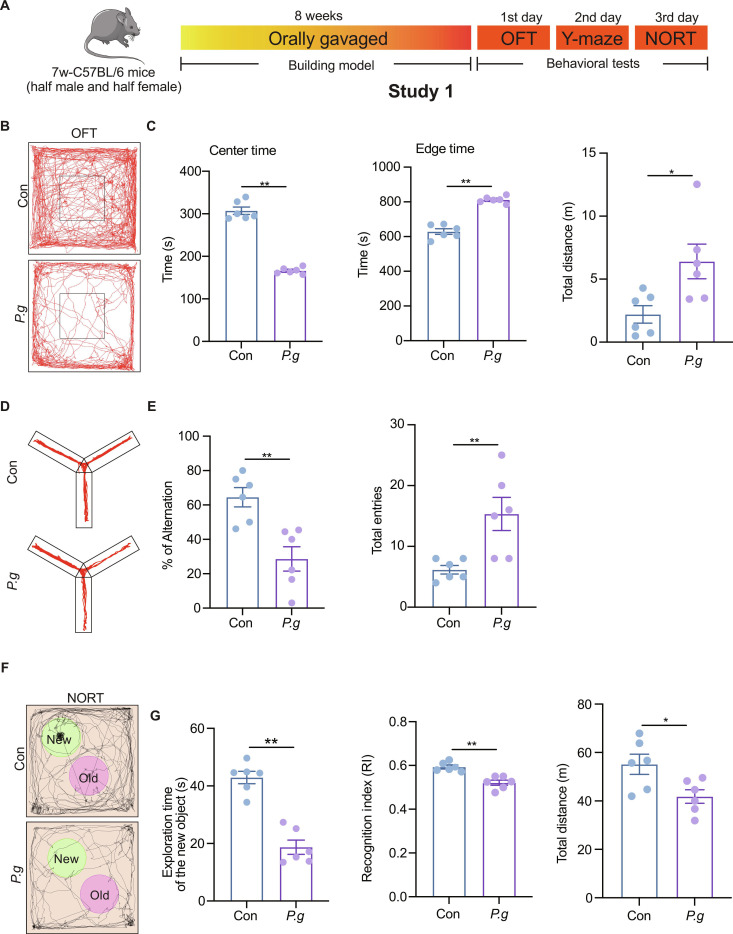
Anxiety degree and cognitive impairment in groups of *Porphyromonas gingivalis* (*P.g*) mice after gavage for 8 weeks. (A) Experimental steps. (B and C) Representative trajectories in OFT, center time, edge time, and total distance of mice moving in OFT. (D and E) Representative trajectories, % of alteration of moving, and total entries of mice (E) in the Y-maze test. (F and G) Representative trajectories, exploration time of mice with the new object, the object discrimination index, and total distance of mice moving in the novel object recognition test (NORT). Two-group comparisons were performed using the unpaired *t* test. Data are presented as the mean ± standard error of the mean (SEM). *P < 0.05, **P < 0.01 versus corresponding controls (Con).

Following the establishment of the *P.g* infection model, mouse behavior was assessed by open field test (OFT), Y-maze, and novel object recognition test (NORT) to evaluate locomotor activity and emotional and cognitive domains (Fig. 1A). *P.g*-infected mice showed signs of increased anxiety-like behavior and hyperlocomotion in OFT, evidenced by reduced center time and increased periphery time (Fig. 1B and C). Spatial working memory, assessed in the Y-maze, was also impaired, with the *P.g* group showing a significantly lower spontaneous alternation rate compared to controls, despite no difference in total arm entries (Fig. 1D and E and Fig. S2A and B). Furthermore, in the NORT, *P.g*-infected mice exhibited impaired novel object recognition and displayed a significantly reduced recognition index, indicating impaired recognition memory (Fig. 1F and G and Fig. S2C and D). Collectively, these behavioral findings demonstrated that *P.g* induces significant anxiety-like behavior and cognitive deficits in mice.

### *P.g* triggers p-Tau and elevates neuroinflammation in hippocampus in vivo

**Fig. 2.**
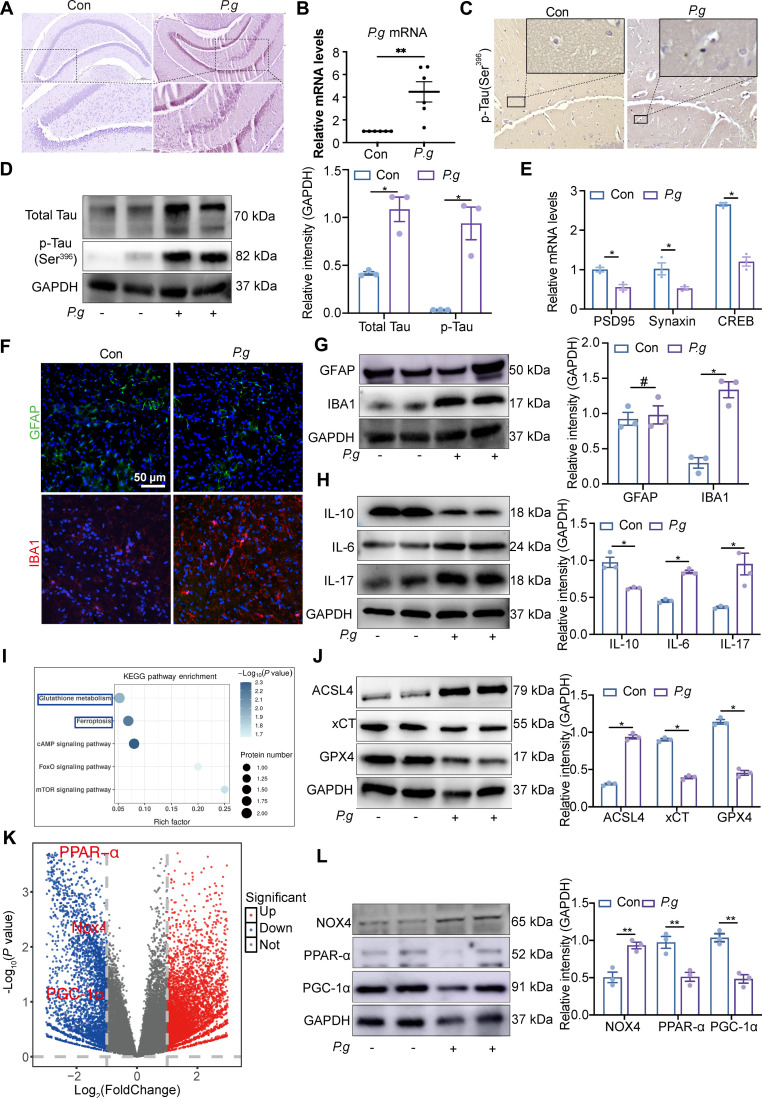
P-Tau accumulation ferroptosis and neuroinflammation in mice (A and B). (A) Immunohistochemical (IHC) images of *Porphyromonas gingivalis* (*P.g*) in the brain tissue. (B) Relative mRNA expressions of *P.g* in the brain tissue. (C) IHC images of p-Tau in the brain tissue. (D) Western blotting images and quantitative analysis of p-Tau in the hippocampus. (E) Relative mRNA expressions of CREB, PSD95, and syntaxin in the brain tissue (*n* = 3). (F) Immunofluorescence images of microglia (IBA1) and astrogliosis (GFAP). (G and H) Western blotting images and quantitative analyses of the expression levels of GFAP, IBA1, interleukin-10 (IL-10), IL-17, and IL-6 in microglia (*n* = 3). (I) Bubble diagram of the Kyoto Encyclopedia of Genes and Genomes (KEGG) enrichment analysis between Con and *P.g* groups. The horizontal axis represented the gene ratio, and the size of dots represented the number of genes in the KEGG term. (J) Western blotting images and quantitative analyses of ACSL4, xCT, and GPX4 in the brain tissue (*n* = 6). (K) Visualization of differentially expressed genes in the brain tissue of mice (*n* = 3). (L) Western blotting images and quantitative analyses of NOX4, PPAR-α, and PGC-1α in the brain tissue (*n* = 3). Two-group comparisons were performed using the unpaired *t* test. Data are presented as the mean ± standard error of the mean (SEM). *P < 0.05, **P < 0.01 versus corresponding controls (Con).

Comparative analysis revealed detectable levels of *P.g* in the brain tissue of infected mice, which was absent in controls (Fig. 2A and B). In light of the hippocampus’s established significance in memory and the progression of AD, key molecular changes were examined in this region following *P.g* infection. A significant up-regulation of P-Tau protein level was observed in the hippocampus of *P.g* group compared with controls (Fig. 2C and D), suggesting enhanced tau pathology. Furthermore, transcript levels of key synaptic regulators (CREB, syntaxin, and PSD-95) were markedly down-regulated in *P.g*-infected mice (Fig. 2E and Fig. S2A). Taken together, this work established a mechanistic link between *P.g* infection and early AD-like pathology, evidenced by promoted Tau hyperphosphorylation and compromised synaptic function in the hippocampus.

Neuroinflammation is a well-established contributor to neuronal damage in AD, a process predominantly fueled by activated microglia and astrocytes through their release of proinflammatory cytokines. Consistent with this notion, we observed a significant increase in IBA1 protein levels, a marker of microglial activation, in the hippocampus of *P.g*-infected mice compared to controls, whereas glial fibrillary acidic protein (GFAP) expression, indicative of astrocyte reactivity, was up-regulated and not significantly changed after statistical analyses (Fig. 2F and G and Fig. S2B). Immunofluorescence costaining revealed a marked decrease in GPX4 signal specifically within Iba1-positive microglia (Fig. S6E), indicating the induction of ferroptosis in microglia. Further supporting a pro-inflammatory shift, cytokine analysis revealed a marked elevation in the levels of interleukin-6 (IL-6), IL-17, IL-1β, and tumor necrosis factor-α (TNF-α), accompanied by a significant reduction in IL-10 in the *P.g* group relative to controls (Fig. 2H and Fig. S2C). Together, these data indicated that *P.g* infection induces microglial activation and skews the hippocampal cytokine milieu toward a pro-inflammatory state, implicating microglia-driven neuroinflammation as a consequential response to periodontal pathogen challenge.

We systematically interrogated the mechanisms of *P.g*-induced hippocampal damage via RNA sequencing. Kyoto Encyclopedia of Genes and Genomes (KEGG) enrichment analysis of the differentially expressed genes demonstrated that glutathione (GSH) metabolism and ferroptosis pathways were prominently disrupted (Fig**.** 2I). Consistent with the transcriptomic data, hippocampal lysates from *P.g*-infected mice exhibited up**-**regulation of ACSL4, along with down**-**regulation of xCT and GPX4 in protein and mRNA levels (Fig. 2J and Fig. S2D). Together, these multi-omics results indicated that *P.g* infection not only triggered microglia-mediated neuroinflammation but also promoted hippocampal ferroptosis, likely through dysregulation of the GSH metabolic pathway.

### *P.g* potentiates neuroinflammation through microglial ferroptosis driven by mitochondrial metabolism in vitro

**Fig. 3.**
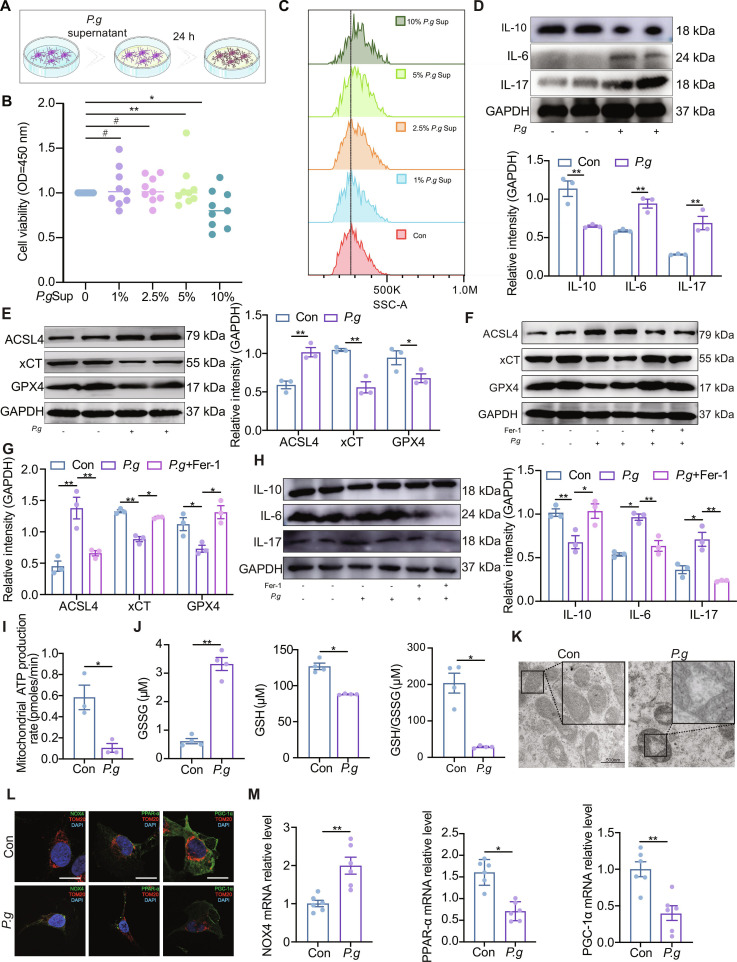
*P.g* promotes microglial mitochondria, ferroptosis, and neuroinflammation. (A) Illustration of *P.g* damage in microglia. (B) CCK-8 results showing the viability of HMC3 cells treated with *P.g* supernatant. (C) Flow cytometry analysis of mitochondrial reactive oxygen species (mtROS) expression in HMC3 cells. (D) Western blotting images and quantitative analyses of the expression levels of interleukin-10 (IL-10), IL-17, and IL-6 in microglia (*n* = 3). (E) Western blotting images and quantitative analyses of the expression levels of ferroptosis in microglia (*n* = 3). (F to H) Western blot analysis of inflammation and ferroptosis level under Fer-1 or *P.g* supernatant treatment in HMC3 cells. (I) Quantification of mitochondrial ATP production rate in microglia. Data are mean ± standard error of the mean (SEM) (*n* = 3). (J) Quantification of reduced oxidized glutathione (GSSG) levels, glutathione (GSH) levels, and ratio of GSH/GSSG in microglia (*n* = 4). (K) Electron microscopy images affected the mitochondrial ultrastructure. (L) Costaining of NOX4, PPAR-α, and PGC-1α with TOM20 in microglia. (M) Relative mRNA expressions of NOX4, PPAR-α, and PGC-1α in HMC3 (*n* = 6). Two-group comparisons were performed using the unpaired *t* test. Multi-group comparisons were performed using one-way analysis of variance (ANOVA). Data are presented as the mean ± SEM. *P < 0.05, **P < 0.01 versus corresponding controls (Con).

To evaluate the dose-dependent effects of *P.g* on microglial activation, HMC3 cells were subjected to a range of *P.g* supernatant concentrations (Fig. 3A and B). Exposure to 5% *P.g* supernatant significantly induced mtROS accumulation (Fig. 3C) and shifted the cytokine profile toward a pro-inflammatory state, characterized by elevated levels of IL-6, IL-17, IL-1β, and TNF-α, along with a reduction in IL-10 (Fig. 3D and Fig. S3A).

Consistent with a ferroptotic response, *P.g* stimulation increased ACSL4 expression while decreasing xCT and GPX4 levels in HMC3 cells (Fig. 3E and Fig. S2B). Importantly, the ferroptosis inhibitor ferrostatin-1 (Fer-1) attenuated *P.g*-induced inflammatory cytokine release (Fig. 3F to H and Fig. S3C and D). These findings collectively demonstrated that the *P.g* supernatant activated pro-inflammatory responses in microglia and induced key ferroptotic alterations, while ferroptosis inhibition mitigated the inflammatory output, supporting a role for ferroptosis in driving *P.g*-triggered neuroinflammation in vitro.

Ferroptosis is closely associated with characteristic mitochondrial alterations, including fragmentation and cristae enlargement. We assessed mitochondrial metabolic function in HMC3 microglia after *P.g* supernatant stimulation. It was observed that *P.g* treatment significantly increased oxidized glutathione (GSSG) levels, reduced adenosine triphosphate (ATP) production, and decreased the GSH/GSSG ratio (Fig. 3I and J), indicating severe oxidative stress and redox imbalance. Consistent with this, transmission electron microscopy (TEM) revealed marked mitochondrial shrinkage and loss of cristae structure in the *P.g*-treated group (Fig. 3K). *P.g* stimulation impaired mitochondrial integrity in HMC3 cells, as evidenced by reduced levels of multiple (electron transport chain complexes and the outer membrane protein TOM20 (Fig. S3E), collectively indicating *P.g*-induced mitochondrial dysfunction.

Based on our RNA-sequencing (RNA-seq) analysis, which highlighted significant perturbations in GSH metabolism (Fig. 2I), several key regulators (NOX4, PPAR-α, and PGC-1α) were detected (Fig. 2L). Both in vivo (Fig. S3E) and in vitro (Fig. 3M), *P.g* infection increased NOX4 expression while decreasing PPAR-α and PGC-1α levels. Importantly, immunofluorescence staining confirmed the colocalization of NOX4, PPAR-α, and PGC-1α with the mitochondrial marker TOM20 (Fig. 3L). These findings collectively suggest that *P.g* impairs microglial mitochondrial metabolism and promotes ferroptosis, likely through a mechanism involving the dysregulation of the NOX4/PPAR-α/PGC-1α axis.

### *P.g* induced neuroinflammation and microglial ferroptosis by impairing mitochondrial function through the NOX4/PPAR-α/PGC-1α signaling pathway

**Fig. 4.**
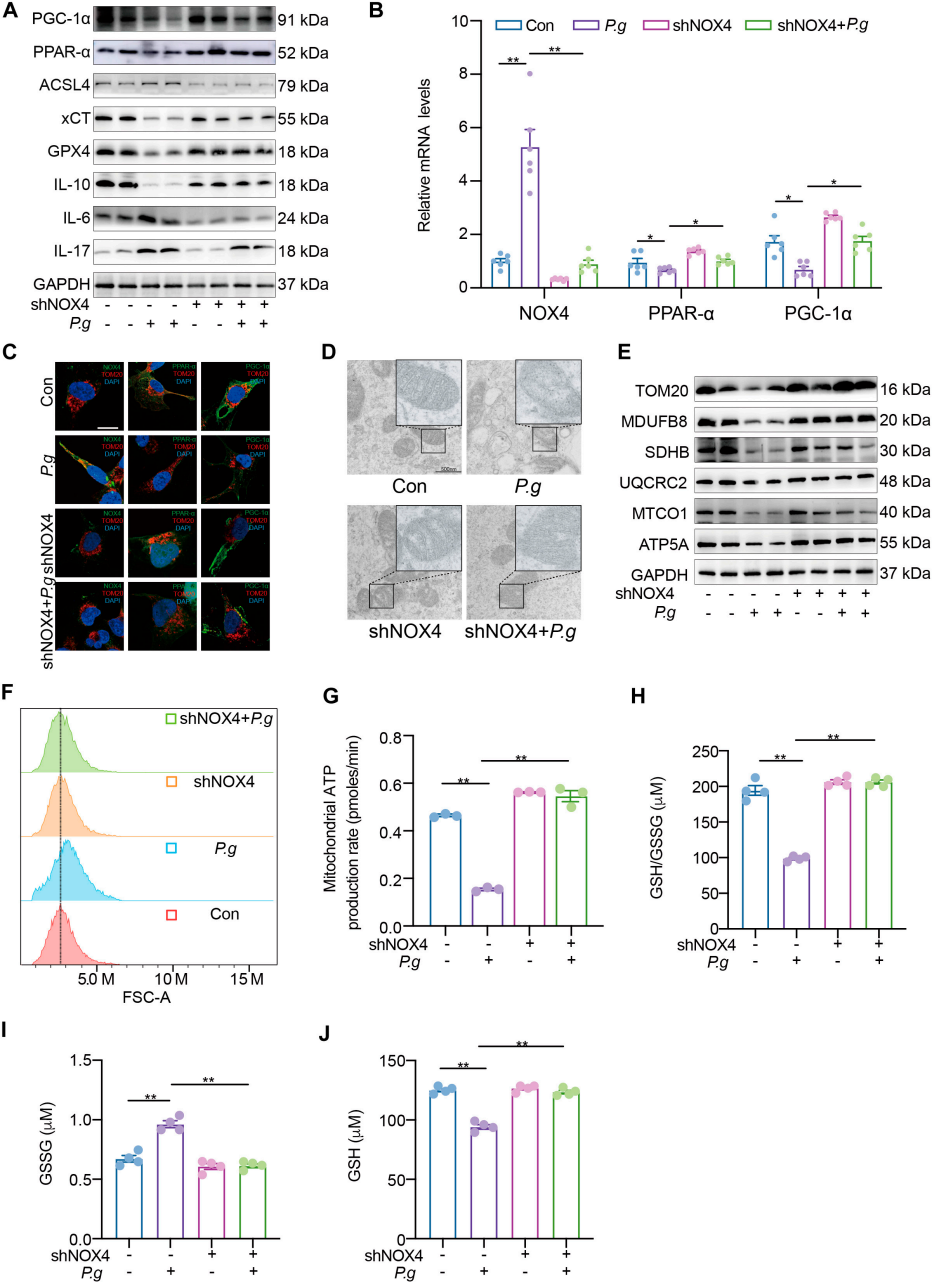
ShNOX4 protects mitochondrial metabolism in HMC3. (A) Western blotting images for PPAR-α, PGC-1α, ferroptosis, and inflammation in microglia. (B) Relative mRNA expressions of NOX4, PPAR-α, and PGC-1α in HMC3 (*n* = 6). (C) Costaining of NOX4, PPAR-α, and PGC-1α with TOM20 in microglia. (D) Transmission electron microscopy image. (E) Western blotting images and quantitative analyses of mitochondrial complex in HMC3 (*n* = 3). (F) Flow cytometry analysis of mitochondrial reactive oxygen species (mtROS) expression in HMC3 cells. (G) Quantification of mitochondrial ATP production rate in microglia (*n* = 3). (H to J) Quantification of reduced glutathione (GSH) levels, oxidized glutathione (GSSG) levels, and ratio of GSH2/GSSG in the control (Con) and *Porphyromonas gingivalis* (*P.g*) groups in microglia (*n* = 4). Multi-group comparisons were performed using one-way analysis of variance (ANOVA). Data are presented as the mean ± standard error of the mean (SEM). *P < 0.05, **P < 0.01 versus corresponding controls. shNOX4, NOX4 post-transfection; shNOX4 + *P.g*, NOX4 post-transfection + *Porphyromonas gingivalis*. Multi-group comparisons were performed using one-way ANOVA. Data are presented as the mean ± SEM. *P < 0.05, **P < 0.01 versus corresponding controls.

**Fig. 5.**
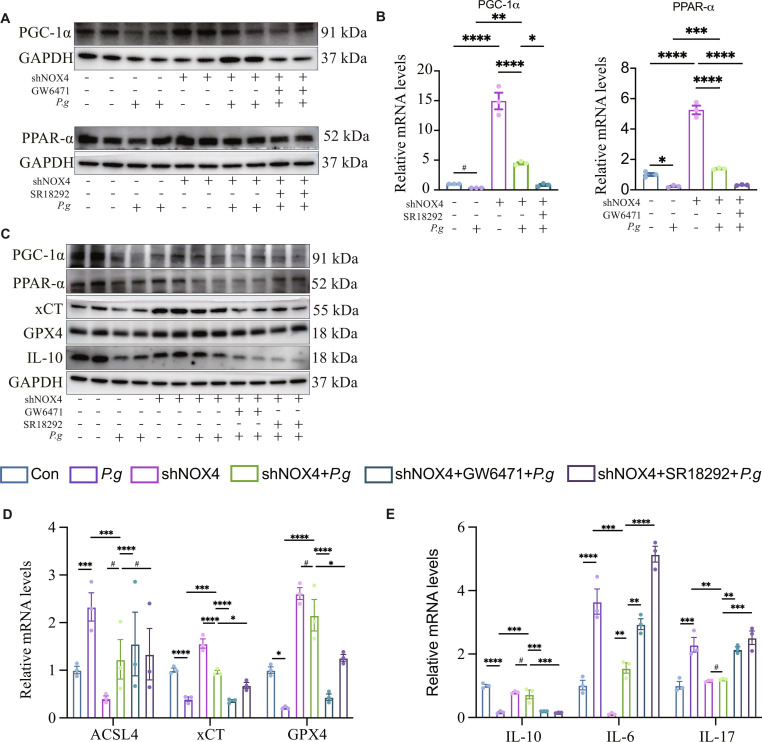
Verification of the upstream and downstream relationships in the NOX4/PPAR-α/PGC-1α pathway in HMC3. (A and B) Expression levels of pathway proteins and mRNA. (C to E) Protein and mRNA levels of PPAR-α, PGC-1-α, ferroptosis, and inflammatory factors. Multi-group comparisons were performed using one-way analysis of variance (ANOVA). Data are presented as the mean ± standard error of the mean (SEM). *P < 0.05, **P < 0.01 versus corresponding controls (Con). *P.g*, *Porphyromonas gingivalis*; shNOX4, NOX4 post-transfection; shNOX4 + *P.g*, NOX4 post-transfection + *Porphyromonas gingivalis*; shNOX4 + GW6471 + *P.g*, NOX4 post-transfection + inhibitor of PPAR-α + *Porphyromonas gingivalis*; shNOX4 + SR18292 + *P.g*, NOX4 post-transfection + inhibitor of PGC-1α + *Porphyromonas gingivalis*

To determine whether *P.g* impairs microglial mitochondrial metabolism via the NOX4/PPAR-α/PGC-1α axis, we knocked down NOX4 in HMC3 cells (Fig. 4A and B and Fig. S4A and B). NOX4 knockdown effectively ameliorated *P.g*-induced ferroptosis and neuroinflammatory responses. Complementary gain-of-function experiments confirmed the sufficiency of NOX4 in this process. Lentiviral-driven overexpression of NOX4 in HMC3 was sufficient to up-regulate pro-inflammatory mediators and induce ferroptosis, as evidenced by elevated lipid peroxides and reduced GPX4 levels (Fig. S6B to D). Furthermore, it reduced the mitochondrial localization of PPAR-α while enhancing that of PGC-1α (Fig. 4C). TEM revealed that NOX4 knockdown restored mitochondrial ultrastructure, attenuating *P.g*-induced shrinkage, cristae loss, and round-shaped morphology (Fig. 4D). The expression of mitochondrial electron transport chain complexes, which was suppressed by *P.g*, was also recovered following NOX4 knockdown (Fig. 4E). Consistent with these morphological and molecular improvements, NOX4 knockdown mitigated *P.g*-induced mitochondrial dysfunction, as evidenced by reduced mtROS and GSSG levels, increased ATP production, and an elevated GSH/GSSG ratio (Fig. 4F to J and Fig. S4C). These results collectively demonstrate that *P.g* disrupts mitochondrial function and metabolism in microglia in a NOX4-dependent manner. To elucidate whether PPAR-α/PGC-1α function as protectors or promoters of ferroptosis and to clarify their role in NOX4 signaling, the ferroptotic phenotype and inflammatory cytokine profiles were examined following treatment with PPAR-α or PGC-1α inhibitors in a shNOX4 background. All experimental results indicated that both Nox4 knockdown and inhibition of PPAR-α/PGC-1α signaling exerted protective effects in the *P.g*-treated group (Fig. 5). These results indicate that NOX4 functions upstream of the PPAR-α/PGC-1α axis.

Together, these findings indicated that *P.g* triggers microglial neuroinflammation and ferroptosis through a regulatory pathway involving NOX4, PPAR-α, and PGC-1α, wherein NOX4 acted upstream of PPAR-α and PGC-1α to disrupt mitochondrial metabolism and promote inflammatory injury.

### NOX4 knockout abolishes *P.g-*induced neuroinflammation and consequently rescues cognitive impairment in vivo

**Fig. 6.**
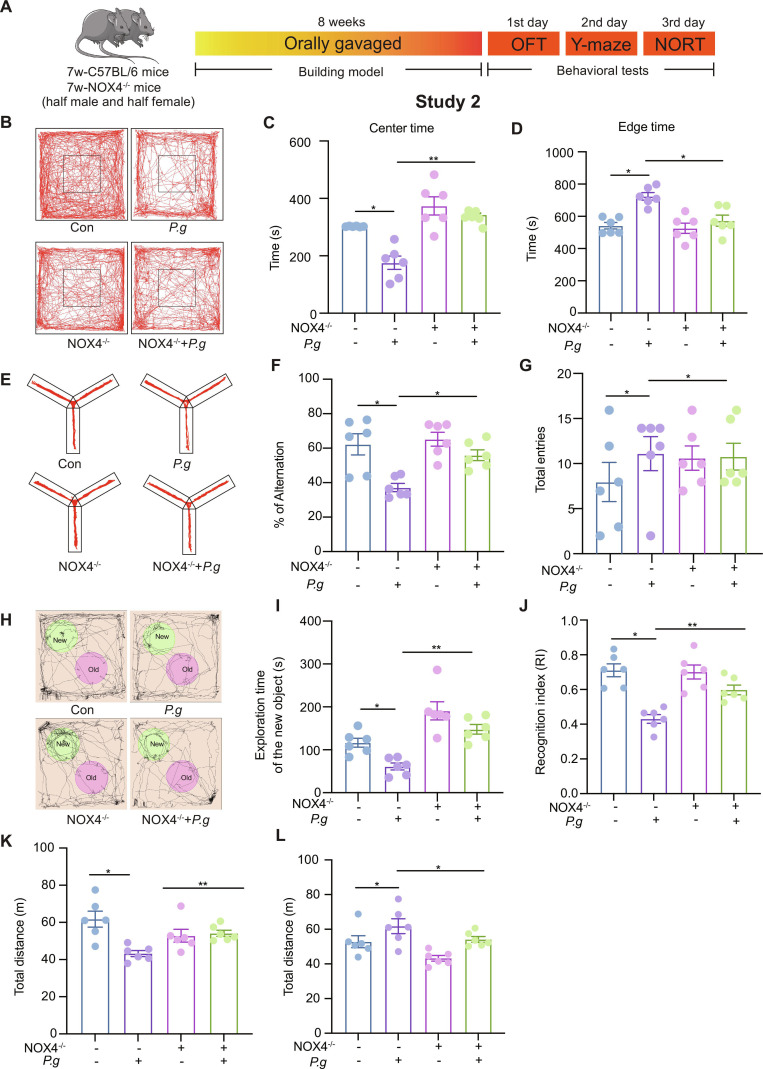
Anxiety degree and cognitive impairment in groups of *P.g* mice after gavage for 8 weeks. (A) Experimental steps. (B to D and L) Representative trajectories in OFT, center time, edge time, and total distance of mice moving in OFT. (E to G) Representative trajectories, % of alteration of moving, and total entries of mice in the Y-maze test. (H to K) Representative trajectories, exploration time of mice with the new object, the object discrimination index, and total distance of mice moving in the novel object recognition test (NORT). Multi-group comparisons were performed using one-way analysis of variance (ANOVA). Data are presented as the mean ± standard error of the mean (SEM). *P < 0.05, **P < 0.01 versus corresponding controls. Con, control; P.g, *Porphyromonas gingivalis*; NOX4^−/−^, *NOX4*-knockout; NOX4^−/−^ + *P.g*, NOX4 -knockout + *Porphyromonas gingivalis*.

To further validate the role of NOX4 in *P.g*-induced neuroinflammation and cognitive deficits in vivo, we subjected *Nox4*-knockout (NOX4^−/−^) mice to an 8-week *P.g* infection regimen (Fig. 6A). OFT revealed that NOX4 deficiency provided a protective effect against *P.g*-induced anxiety-like behavior, as evidenced by increased center time, decreased periphery time, and reduced hyperlocomotion compared to the infected WT group (Fig. 6B to D and L), indicating attenuated anxiety-like behavior. Spatial working memory, assessed in the Y-maze, was also improved, as shown by a higher spontaneous alternation rate in the NOX4^−/−^ + *P.g* group (Fig. 6E to G). Furthermore, during the NORT, these mice showed a clear preference for the novel object, with significantly increased exploration time and number of visits, demonstrating rescue of *P.g*-induced learning and memory impairment (Fig. 6H to K).

**Fig. 7.**
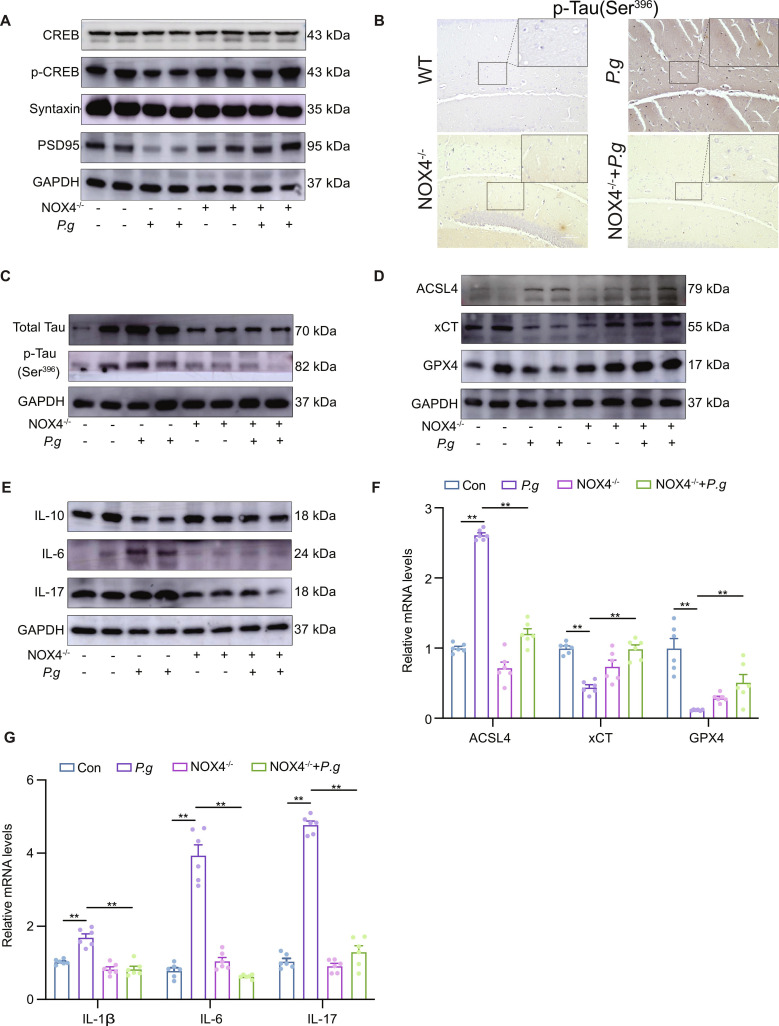
NOX4^−/−^ salvages the *P.g* -induced ferroptosis, neuroinflammation, and pathologies. (A) Western blotting images of CREB, p-CREB, syntaxin, and PSD-95 in brain tissues. (B) Representative images of p-Tau immunohistochemistry. (C to E) Western blotting images of hippocampal Tau, p-Tau, ferroptosis, and inflammation in brain tissues. (F and G) Relative mRNA expressions of ferroptosis and inflammation in brain tissues. Multi-group comparisons were performed using one-way analysis of variance (ANOVA). Data are presented as the mean ± standard error of the mean (SEM). *P < 0.05, **P < 0.01 versus corresponding controls. Con,control; P.g, *Porphyromonas gingivalis*;NOX4^−/−^, *NOX4*-knockout; NOX4^−/−^ + *P.g*, *NOX4*-knockout + *P.g*.

At the molecular level, Nox4 knockout reversed *P.g*-induced synaptic deficits, elevating the expression of PSD-95, syntaxin, and CREB while reducing phosphorylated Tau levels in the hippocampus (Fig. 7A to C and Fig. S5A to D). It also suppressed ferroptosis, as evidenced by decreased ACSL4 and increased xCT and GPX4 expression (Fig. 7D and Fig. S5E). Consistently, NOX4^−/−^ + *P.g* mice showed a marked decrease in IL-6 and IL-17, along with an elevation of the anti-inflammatory cytokine IL-10 (Fig. 7G and Fig. S5F).

In summary, our in vivo data demonstrate that genetic ablation of NOX4 alleviates *P.g*-induced hippocampal synaptic dysfunction, cognitive impairment, and neuroinflammation, likely through inhibition of ferroptosis, highlighting NOX4 as a critical mediator in periodontal pathogen-driven neurodegeneration.

## Discussion

Neuroinflammation is a complex cascade, triggered by cerebral immune activation in response to injury, infection, or disease stimuli. This process drives the release of proinflammatory mediators, including cytokines and ROS, ultimately contributing to neuronal death and cognitive decline [24,25]. This study revealed that *P.g* caused cognitive impairment via neuroinflammation in mice. Furthermore, *P.g* affects neuroinflammation by inducing ferroptosis by damaging mitochondrial function in microglia through the NOX4/PPAR-α/PGC-1α pathway in the hippocampus on the neuronal functions, suggesting its potential as a therapeutic target against *P.g* infection and periodontitis.

Oral bacteria, especially *P.g*, is causally linked to the pathophysiology of AD. Studies have shown that *P.g* LPS, DNA, and gingipains were detected in the brains of patients with AD [13,26]. In this study, oral administration of *P.g* in mice induced cognitive impairment, Tau phosphorylation, and neuroinflammation. These findings suggest that immune crosstalk between the oral cavity and the brain plays a critical role in that periodontitis exacerbates AD pathology (Figs. 1 and 2). After *P.g* gavaged in mice, *P.g* gingipains appear in neurons, microglia, and astrocytes [27]. As the disease progresses, activated microglia exert detrimental effects, driven by the overexpression of major inflammatory cytokines, represented by IL-1β, IL-6, and TNF-α [28,29]. Importantly, our study detected an up-regulation of markers specific to microglia in vivo, especially the marker of microglia IBA1, which suggests that microglia activation plays the main role in neuroinflammation after gavage with *P.g*.

AD mice showed that ferroptosis was progressively down-regulated in the hippocampus with age [30]. Ferroptosis accelerates AD pathogenesis by inducing neuronal dysfunction and triggering aberrant microglial activation. This process is fueled by disrupted iron homeostasis, which in turn exacerbates neuroinflammation. The resulting activated microglia release proinflammatory factors that further dysregulate iron metabolism, thereby establishing a self-perpetuating vicious cycle between ferroptosis and neuroinflammation [31]. In our study, RNA sequencing analysis found that the ferroptosis and glutamate metabolism were involved (Fig. 2). Consistent with previous studies, our results found that both ferroptosis and neuroinflammation were induced in vivo and in vitro (Figs. 2 and 3). Given that ROS-driven lipid peroxidation initiates ferroptosis, inhibiting ROS offers a logical therapeutic avenue to counteract this form of cell death [32]. In this study, the ROS level and ferroptosis in HMC3 were significantly up-regulated after treatment with the *P.g* supernatant (Fig. 3). Early research established that glial activation, along with the generation of pro-inflammatory cytokines (e.g., IL-1β, IL-6, and TNF-α) and ROS, collectively contributes to neuronal dysfunction and death [33–35]. In this study, Fer-1 inhibited the *P.g*-induced neuroinflammation factors in HMC3 (Fig. 3), which showed that *P.g* induced neuroinflammation by activation ferroptosis in microglia.

Ferroptosis is linked to mitochondrial structure and function. Mitochondrial dysfunction and impaired mitochondrial oxygen metabolism are recognized features of the hippocampus in AD patients [36]. Alteration in mitochondrial form and function, including metabolic changes, ROS increasing, and decreased ATP production, potentially contributes to ferroptosis [20,37]. Maintaining the mitochondria function reduces the ROS level and alleviates the AD progress. We found that *P.g* stimulation compromised mitochondrial structure and function in HMC3 cells. *P.g* stimulation elicited significant damage to mitochondrial morphology and function in HMC3 cells, and mitochondrial complex proteins were decreased in vitro (Fig. 3), which showed that *P.g* induced neuroinflammation via ferroptosis activation by damaging mitochondria function of HMC3.

NOX4, as a family member of NOXs, is a mitochondrial energy metabolism switch on oxidative stress and ROS production during brain injury, which regulates the mitochondrial structure and function. More recently, it has been reported that NOX2, NOX3, and NOX4 are expressed in microglia [38]. Among these, NOX4 mainly locates in the mitochondria and contains a mitochondria-targeting signal [39]. Elevated NOX4 activity has been linked to cognitive impairment in AD [40]. In our study, *P.g* up-regulated NOX4 in HMC3. After the NOX4 knockdown in HMC3, the morphology and protein expression levels of mitochondria, the ROS level, and the metabolic function were rescued. Meanwhile, NOX4 knockdown rescued ferroptosis and neuroinflammation in HMC3, and cognitive impairment in vivo after *P.g* stimulation (Figs. 4, 6, and 7). These showed that *P.g* caused cognitive impairment via neuroinflammation, which was dependent on ferroptosis activation by damaging the mitochondria through NOX4 up-regulation in microglia.

Peroxisome proliferator-activated receptors (PPARs) are a family of ligand-activated nuclear receptors, comprising 3 subtypes: PPAR-α, PPAR-β/δ, and PPAR-γ [41]. PPAR-α is a key regulator of mitochondrial metabolism, broadly governing fatty acid β-oxidation, energy production, glucose metabolism, and redox homeostasis. Additionally, it modulates key neurotransmission systems, including glutamatergic, cholinergic, and dopaminergic pathways [42]. PPAR-α may modulate Tau phosphorylation through multiple pathways, either directly or by regulating the cerebral metabolism of Aβ [41]. Elevated NOX4 levels promote the generation of mtROS, which down-regulates PPAR-α and leads to mitochondrial breakdown and cellular oxidation processes [43]. The activation of PPARs can stimulate the PGC-1α pathway, a key regulator of mitochondrial health. PGC-1α promotes mitochondrial biogenesis by enhancing mitochondrial transcription, induces uncoupling proteins and antioxidant enzymes, and mitigates oxidative stress. The observed down-regulation of PGC-1α in AD hippocampi is likely a significant contributor to the disease’s characteristic mitochondrial impairment [44,45]. The *P.g* group recapitulated the key RNA-seq findings: elevated NOX4 and suppressed PPAR-α/PGC-1 in both animal and cell-based experiments (Figs. 2 and 3). More importantly, NOX4, PPAR-α, and PGC-1a were colocalized in the mitochondria in HMC3. The down-regulation of PPAR-α and PGC-1α, ferroptosis, and neuroinflammation induced by *P.g* in HMC3 were rescued after NOX4 knockdown (Fig. 4). However, the inhibitor of PPAR-α and PGC-1α blocked the protection role of NOX4 knockdown in HMC3 from ferroptosis and neuroinflammation after *P.g* infection*.* The inhibitors of PPAR-α and PGC-1α rescued ferroptosis and neuroinflammation induced by *P.g* in HMC3 after NOX4 knockdown (Fig. 5)*.* Our results implicated that *P.g* induced neuroinflammation and ferroptosis in HMC3 by maintaining the mitochondrial function by activating the NOX4/PPAR-α/PGC-1α pathway. Given the current challenges in periodontal disease management, this study prompts important clinical considerations regarding whether improved oral hygiene measures targeting *P.g* reduction could potentially lower AD risk. Additionally, the therapeutic potential of combining NOX4 inhibitors with existing AD treatments warrants exploration. However, there are some limitations. Firstly, given the systemic dissemination of *P.g* to multiple organs, it is imperative to consider its potential to exacerbate AD through alternative pathways, such as gut–brain axis communication [46], interactions with astrocytes [47], and so on. Our findings suggest that ferroptosis activation serves as a critical link between *P.g* infection and neuroinflammation, primarily through the release of damage-associated molecular patterns (DAMPs). The ferroptotic process, characterized by mitochondrial dysfunction and lipid peroxidation, leads to the leakage of intracellular components such as mitochondrial DNA and HMGB1. These DAMPs can subsequently activate pattern recognition receptors [e.g., Toll-like receptors (TLRs) and stimulator of interferon genes (STING)] on microglia, triggering the production of pro-inflammatory cytokines and establishing a self-amplifying cycle of inflammation and cellular damage. This mechanism provides a plausible pathway through which peripheral infection drives CNS pathology. Secondly, this study utilized the *P.g* supernatant, rather than the live bacterium, to stimulate microglia and explore the underlying molecular mechanisms, which presents limitations in identifying specific virulence determinants. Subsequent studies will employ purified bacterial factors or genetically modified knockout strains to pinpoint critical pathogenic molecules. Finally, this study focused on direct neuroinflammatory pathways. Potential indirect mechanisms (e.g., gut–brain axis involvement) remain plausible. Future investigations could utilize gnotobiotic models or intestinal barrier integrity assessments to exclude secondary pathways. Consequently, the specific virulence factors of *P.g* responsible for promoting AD pathogenesis remain to be identified in the future.

## Materials and Methods

### Mice and study design

Six-week-old WT and *Nox4*-knockout C57BL/6 mice were housed under specific pathogen-free conditions. Following 1-week acclimatization period to minimize aggression, the mice were subjected to an 8-week gavage regimen. Behavioral assays were performed during the light cycle. Upon completion of these tests, brain tissues were harvested and stored at −80 °C for subsequent analysis [48]. The animal experiments were approved by the Animal Ethics Committee of the Tenth People’s Hospital of Tongji University (ethics nos. SHDSYY-2022-3063-21 and SHDSYY-2022-3063-22). Study 1 investigated the effect of *P.g* on cognition-like behavior, whereas study 2 validated its critical role following Nox4 knockout. The behavioral testing protocols for both studies are outlined below.

#### Study 1: *P.g* infection model in WT mice

Sixteen WT male and female mice were randomly distributed into 2 groups (*n* = 8 per group): a control group (Con) gavaged with sterile Brain Heart Infusion (BHI) broth, and a *P.g* gavage group (*P.g* group) gavaged with live *P.g* [10^9^ colony-forming units (CFU) suspended in 200 μl of BHI; Beijing Solarbio Science & Technology Co. Ltd.]. Following an 8-week regimen of daily oral broth administration, all mice underwent behavioral testing. Food and water were available ad libitum for the duration of the study. The experimental timeline is detailed in Fig. 1A.

#### Study 2: *P.g* infection model in *Nox4*-knockout mice

Sixteen WT and 16 *Nox4*-knockout C57BL/6 mice were randomly distributed among 4 groups (*n* = 8 per group, 50% male): BHI gavaged group (Con group), *P.g* gavaged group (*P.g* group), *Nox4*^−/−^ + BHI group (*Nox4*^−/−^ group), and *Nox4*^−/−^ + *P.g* (*Nox4*^−/−^ + *P.g* group). During the 8-week daily oral gavage regimen, all animal groups had unrestricted access to food and water. Behavioral tests were conducted upon completion of the gavage regimen. The experimental timeline is shown in Fig. 6A.

### Mouse behavioral tests

A series of behavioral tests (OFT, Y-maze, and NORT) were employed to assess cognitive and memory function following an acclimation period. Testing was conducted under standard lighting, with inter-trial cleaning of apparatus using 70% ethanol to prevent olfactory bias.

### OFT

The trial commenced by placing a mouse in the central arena, oriented toward an open arm, and allowing it to explore freely for 5 to 10 min while its activity was video-tracked. The primary metric, open arm residence time, was quantified as a percentage of the total session duration.

### Y-maze

The Y-maze, consisting of 3 symmetrical arms (40 cm × 8 cm × 15 cm), was used for a 5-min free exploration trial. Arm entries were recorded to calculate the percentage of spontaneous alternation (defined as consecutive entries into all 3 arms) and the total number of entries.

### NORT

NORT was performed over 3 d: habituation to an empty arena, training with 2 identical objects, and a final test where one object was replaced with a novel one. Each session lasted 5 to 10 min. From the test session, we quantified novel object exploration time, the recognition index, and total distance moved.

### Bacterial cultures

*P.g* [strain ATCC (American Type Culture Collection) 33277] was primarily cultured on Columbia Agar Base under anaerobic conditions at 37 °C for 3 d. A single colony was then subcultured in BHI broth, supplemented with vitamin K (0.5 μg/ml), protohemin (5 μg/ml), yeast extract (1 mg/ml), and cysteine (0.05%). Bacterial cells were harvested by centrifugation at 3,000 rpm for 5 min, and the resulting supernatant was collected and diluted with cell culture medium for subsequent experiments.

### Cell culture and treatment

HMC3 human microglia (ATCC CRL-3304) were maintained in α-minimum essential medium (MEM) (Gibco) supplemented with 10% fetal bovine serum (FBS; Gibco). At approximately 80% confluence, cells were switched to serum-free medium prior to experimental treatments. For stimulation, cells were exposed to *P.g* supernatant for 24 h. In experiments involving shNOX4 gene cells and utilizing PPAR-α inhibitor (GW6471: 10 μM) or PGC-1α inhibitor (SR18292: 10 μM), shNOX4 cells were pretreated with inhibitors for 24 h prior to *P.g* supernatant challenge.

### Lentiviral transfection

HMC3 cells were seeded in 60-mm dishes and transfected at 60% to 80% confluence using the manufacturer’s recommended protocol. Following a 72-h incubation, successfully transfected cells were harvested and prepared for analysis. Protein and RNA samples were collected for Western blot, polymerase chain reaction (PCR), and immunofluorescence assays.

### Cell viability analysis and inhibitor studies

We evaluated cell viability using the Cell Counting Kit-8 (CCK-8) assay and live/dead cell staining. In the CCK-8 assay, we seeded HMC3 cells into 96-well plates (5 × 10^4^ cells per well) and allowed them to adhere overnight. The culture medium was then replaced with α-MEM (supplemented with 10% FBS) containing serial concentrations (1% to 10%) of *P.g* supernatant for 24 h. After the treatment, we added 10 μl of CCK-8 reagent to each well, incubated the plates at 37 °C for 90 min, and finally measured the absorbance at 450 nm. For the follow-up experiments, the culture medium consisted of α-MEM supplemented with 10% FBS and 5% (v/v) supernatant from *P.g* (at a concentration of 10^9^ CFU/ml).

### Mitochondrial ATP production rate assay

Real-time mitochondrial ATP production kinetics in HMC3 cells were determined using the Seahorse XF Real-Time ATP Rate Assay Kit on an XF96e bioanalyzer (both from Agilent Technologies). In brief, cells were plated at 5 × 10^4^ cells per well in specialized XF96 microplates, and the assay was carried out as per the manufacturer’s guidelines.

### Measurement of GSH, GSSG, and GSH/GSSG ratio

After seeding HMC3 cells in 6-well plates (2 × 10^5^ cells per well) and treating them with *P.g* supernatant for 24 h, we measured GSH levels. Specifically, the concentrations of total GSH and GSSG, as well as their ratio, were determined with GSSG/GSH Quantification Kit II per the manufacturer’s instructions.

### Measurement of mtROS levels

We detected mtROS using the fluorescent probe HPF (MCE, HY-111330). HMC3 cells (2 × 10^5^ cells per well) were stained with 5 μM HPF (from a 10 mM dimethyl sulfoxide stock) in phosphate-buffered saline (PBS) for 30 min at 37 °C in the dark, then trypsinized, resuspended, and immediately analyzed by flow cytometry.

### Immunofluorescence assays

All samples were collected between 08:00 AM and 12:00 PM to minimize circadian variability. We anesthetized mice with isoflurane and performed transcardial perfusion with ice-cold PBS and 4% paraformaldehyde (PFA). The brains were then postfixed in 4% PFA at 4 °C overnight, cryoprotected in a graded sucrose series (20% and 30%), embedded in optimal cutting temperature compound (OCT), and cryosectioned at 25 μm.

For immunofluorescence, brain sections and HMC3 cells were processed in parallel. Both were fixed in 4% PFA (20 min for cells), permeabilized with 0.3% Triton X-100, and blocked with 5% bovine serum albumin for 1 h (sections) or 30 min (cells). Samples were incubated overnight at 4 °C with the following primary antibodies: rabbit anti-GPX4 (1:1,000, A13309), rabbit anti-NOX4 (1:1,000, ABclonal, A22149), mouse anti-PPAR-α (1:1,000, Proteintech, 66826-1-Ig), mouse anti-PGC-1α (1:1,000, Proteintech, 66369-1-Ig), mouse anti-TOM20 (5 μg/ml, Santa Cruz Biotechnology, sc-17764), rabbit anti-IBA1 (1:1,000, Abcam, ab178846), and rabbit anti-GFAP (1:300, ABclonal, A19058).

After PBS washes, samples were processed for immunofluorescence by incubation with Alexa Fluor 488/594 secondary antibodies (2 h, room temperature) and 4′,6-diamidino-2-phenylindole (DAPI) counterstaining. Images were captured with a Nikon fluorescence microscope and analyzed quantitatively using ImageJ software 1.80 (National Institutes of Health, Bethesda, MD, USA) prior to unblinding to the experimental conditions.

### Cellular Fe^2+^ levels

Cellular Fe^2+^ levels were quantified using the FerroOrange probe following the manufacturer’s protocol. Briefly, cells were washed with PBS, incubated with 1 μM FerroOrange in PBS for 30 min and Hoechst 33342 (Thermo Fisher Scientific; catalog no. H3570), 1 μg/ml for 10 min in the dark, and then subjected to measurement.

### Immunohistochemical assays

Brain sections underwent antigen retrieval using citrate buffer (Servicebio, G1202), followed by PBS washes and blocking with serum. Subsequently, sections were incubated overnight with anti-*P.g* (1:100, Developmental Studies Hybridoma Bank, 61BG1.3) and rabbit anti-phospho-Tau (1:500, Cell Signaling Technology, 12885) according to the manufacturer’s protocol. After washing, slices were incubated with the corresponding secondary antibody and developed with 3,3′-diaminobenzidine (DAB; Servicebio, G1211). For each section, 3 representative hippocampal fields were imaged under a Carl Zeiss LSM710 microscope.

### TEM observation

After 24 h of exposure to *P.g* supernatant, HMC3 cells were collected, prefixed in 2.5% glutaraldehyde phosphate (0.1 M, pH 7.4) overnight at 4 °C, and postfixed in 2% buffered osmium tetroxide at 4 °C for 15 min. The fixed cells were dehydrated with 70%, 80%, 90%, and 100% ethanol (each for 15 min). Subsequently, the cells were embedded in Epon812 (Merck KGaA) at room temperature for 30 min, and ultrathin sections (60 nm) were cut and stained with uranyl acetate and lead citrate at room temperature for 30 min. Images were captured using TEM (FEI; Thermo Fisher Scientific Inc.).

### Western blot analysis

Following transcranial perfusion of deeply anesthetized mice, hippocampi were microdissected and divided into hemispheres for parallel protein (Western blot) and RNA [quantitative PCR (qPCR)] analyses, optimizing tissue use for evaluating microglial activity and ferroptosis post-*P.g* infection. All samples were snap-frozen and stored at −80 °C.

Western blot was performed as previously described [26]. Protein lysates from hippocampal tissues were quantified by bicinchonininc acid (BCA) assay following homogenization. Subsequently, equal protein loads were subjected to sodium dodecyl sulfate–polyacrylamide gel electrophoresis (SDS-PAGE) (10%) and transferred to polyvinylidene difluoride membranes. After blocking with 5% nonfat milk, membranes were incubated with primary antibodies (4 °C, overnight) and then with horseradish peroxidase-conjugated secondary antibodies (1:2,000, A0216 or A0208; Beyotime, China) for 1 h at room temperature. The primary antibodies used in Western blotting included rabbit anti-ACSL4 (1:1,000, A20414, ABclonal, China), rabbit anti-GPX4 (1:1,000, A13309, ABclonal, China), rabbit anti-xCT (1:1,000, A2413, ABclonal, China), rabbit anti-IL-6 (1:1,000; A1570, ABclonal, China), rabbit anti-IL-17 (1:200, A10587, ABclonal, China), rabbit anti-IL-10 (1:1,000, A2171, ABclonal, China), rabbit anti-PSD95 (1:1,000, A7889, ABclonal, China), rabbit anti-synaptophysin (1:1,000, A19122, ABclonal, China), rabbit anti-phospho-cyclic adenosine monophosphate response element-binding protein (p-CREB; 1:1,000, AP0019, ABclonal, China), rabbit anti-CREB (1:1,000, ab32515, Abcam, USA), rabbit anti-NOX4 (1:1,000, A22149, ABclonal, China), mouse anti-PPAR-α (1:1,000, 66826-1-Ig, Proteintech, China), mouse anti-PGC-1α (1:1,000, 66369-1-Ig, Proteintech, China), rabbit anti-TOM20 (1:1,000, A19403, ABclonal, China), rabbit anti-ATP5A (1:1,000, A11217, ABclonal, China), rabbit anti-NDUFB8 (1:1,000, A19732, ABclonal, China), rabbit anti-ubiquinol-cytochrome c reductase core protein 2 (UQCRC2) (1:1,000, A4366, ABclonal, China), rabbit anti-succinate dehydrogenase complex iron sulfur subunit B (SDHB; 1:1,000, A23832, ABclonal, China), rabbit anti-MTCO1 (1:1,000, A17889, ABclonal, China), rabbit anti-Tau (1:1,000, A23490, ABclonal, China), rabbit anti-phospho-Tau (1:1,000, 12885, Cell Signaling Technology, USA), rabbit anti-AIF1/IBA1 (1:1,000, A19776, ABclonal, China), rabbit anti-GFAP1 (1:1,000, A19058, ABclonal, China), and rabbit anti-glyceraldehyde-3-phosphate dehydrogenase (GAPDH) (1:1,000, AC001, ABclonal, China). Visualization of immunoblots was achieved employing an ECL Chemiluminescence Kit (P0018S, Beyotime, China), followed by densitometric analysis with ImageJ software 1.80 (National Institutes of Health, USA).

### RNA extraction and qPCR

Total RNA from frozen hippocampal tissues was extracted using a commercial isolation kit (Vazyme Biotech, China), and the concentration and purity were determined using a Nanodrop spectrophotometer (Thermo Fisher Scientific, USA), which was precalibrated with nuclease-free water.

Following reverse transcription of total RNA to cDNA using Ezscript Reverse Transcription Mix II (Takara Bio, Japan), qPCR was conducted in 10-μl reactions comprising cDNA and SYBR Green master mix on a 96/384-well platform. The thermal cycling protocol included initial denaturation (95 °C, 5 min), 40 cycles of 95 °C for 10 s and 60 °C for 30 s, with fluorescence detection at each extension. All reactions were performed in triplicate with GAPDH as the endogenous control, and relative expression was determined via the 2^−ΔΔCt^ method.

### RNA sequencing

Total RNA was extracted from whole-brain tissues and submitted for RNA sequencing (Applied Protein Technology, China). Sequencing libraries were constructed using the Illumina TruSeq RNA Sample Preparation Kit with 1 μg of total RNA as input. Briefly, poly(A)+ mRNA was enriched using oligo(dT) magnetic beads and chemically fragmented. First-strand cDNA was synthesized with random hexamers, followed by second-strand synthesis to generate double-stranded cDNA. The resulting fragments were subjected to end repair, adenylation, and ligation of Illumina adapters. Libraries were size-selected [~300 base pairs (bp)] using 2% low-range ultra-agarose gel electrophoresis and amplified with Phusion DNA polymerase for 15 cycles. After quantification with the TBS380 system, the libraries were sequenced on an Illumina HiSeq X Ten/NovaSeq 6000 platform in paired-end mode (2 × 150 bp). All data processing and bioinformatic analyses were performed using the Applied Protein Technology Cloud Platform (https://bio-cloud.aptbiotech.com/).

### Statistical analyses

All values represent the mean ± standard error of the mean (SEM) of at least 3 biological replicates. Differences between groups were assessed by Student’s *t* test or one-way analysis of variance (ANOVA) using GraphPad Prism (GraphPad Software, California, USA), and a *P* value of less than 0.05 was considered statistically significant.

## Figures and Tables

**Table A1.** Nucleotide sequences of the primers used for reverse transcription PCR

Genes	Forward primer sequence (5’ to 3’)	Reverse primer sequence (3’ to 5’)
*GAPDH(M)*	AGGTCGGTGTGAACGGATTTG	GGGGTCGTTGATGGCAACA
*CREB(M)*	AGCAGCTCATGCAACATCATC	AGTCCTTACAGGAAGACTGAACT
PSD95*(M)*	TCCGGGAGGTGACCCATTC	TTTCCGGCGCATGACGTAG
Syntaxin*(M)*	AGAGATCCGGGGCTTTATTGA	AATGCTCTTTAGCTTGGAGCG
GFAP*(M)*	CGGAGACGCATCACCTCTG	TGGAGGAGTCATTCGAGACAA
IBA1*(M)*	CTTGAAGCGAATGCTGGAGAA	GGCAGCTCGGAGATAGCTTT
*IL-6(M)*	CTGCAAGAGACTTCCATCCAG	AGTGGTATAGACAGGTCTGTTGG
*IL-17(M)*	TCAGCGTGTCCAAACACTGAG	CGCCAAGGGAGTTAAAGACTT
*TNF-α(M)*	CAGGCGGTGCCTATGTCTC	CGATCACCCCGAAGTTCAGTAG
*ACSL4(M)*	CCTGAGGGGCTTGAAATTCAC	GTTGGTCTACTTGGAGGAACG
*xCT(M)*	AGGGCATACTCCAGAACACG	GGACCAAAGACCTCCAGAATG
*GPX4(M)*	TGTGCATCCCGCGATGATT	CCCTGTACTTATCCAGGCAGA
*NOX4(M)*	TGCCTGCTCATTTGGCTGT	CCGGCACATAGGTAAAAGGATG
*PPAR-α(M)*	AACATCGAGTGTCGAATATGTGG	CCGAATAGTTCGCCGAAAGAA
*PGC-1α(M)*	TATGGAGTGACATAGAGTGTGCT	GTCGCTACACCACTTCAATCC
*GAPDH(H)*	GGAGCGAGATCCCTCCAAAAT	GGCTGTTGTCATACTTCTCATGG
*IL-6(H)*	ACTCACCTCTTCAGAACGAATTG	CCATCTTTGGAAGGTTCAGGTTG
*IL-17(H)*	TCCCACGAAATCCAGGATGC	GGATGTTCAGGTTGACCATCAC
*TNF-α(H)*	CCTCTCTCTAATCAGCCCTCTG	GAGGACCTGGGAGTAGATGAG
*ACSL4(H)*	CATCCCTGGAGCAGATACTCT	TCACTTAGGATTTCCCTGGTCC
*xCT(H)*	TCTCCAAAGGAGGTTACCTGC	AGACTCCCCTCAGTAAAGTGAC
*GPX4(H)*	GAGGCAAGACCGAAGTAAACTAC	CCGAACTGGTTACACGGGAA
*NOX4(H)*	CAGATGTTGGGGCTAGGATTG	GAGTGTTCGGCACATGGGTA
*PPAR-α(H)*	ATGGTGGACACGGAAAGCC	CGATGGATTGCGAAATCTCTTGG
*PGC-1α(H)*	TCTGAGTCTGTATGGAGTGACAT	CCAAGTCGTTCACATCTAGTTCA
*P.g*	AGAGTTTGATCCTGGCTCAG	GGGTCTTGTACACACCG

## Data Availability

The data that support the findings of this study are available onreasonable request from the corresponding authors.
