# Supplementary material for: Porphyromonas gingivalis Promotes Neuroinflammation by Microglial Ferroptosis via NOX4/PPAR-α/PGC-1α Pathway
Source: Research (Wash D C). 2026 Apr 8;9:1163. doi: 10.34133/research.1163 (PMC13058221; doi:10.34133/research.1163)
Supplement: Supplementary 1 — Figs. S1 to S6 [file research.1163.f1.zip › Supplement Figure 1.docx]

**Supplement Figure 1**

**
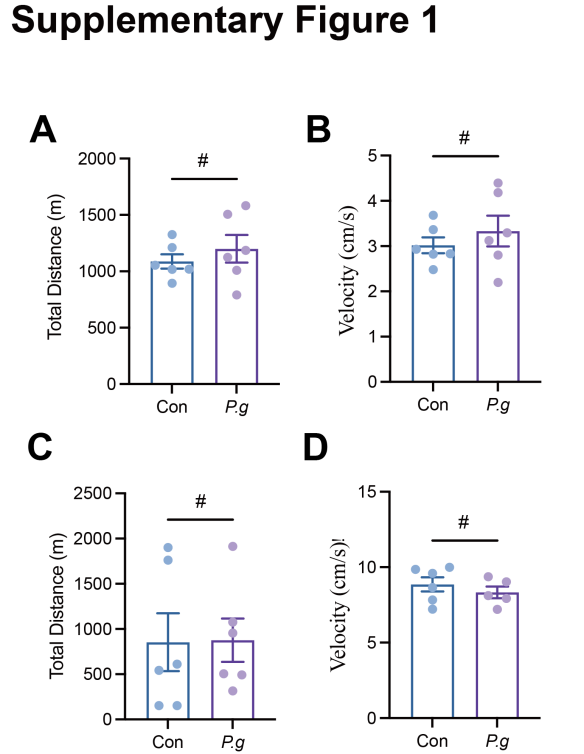
**

Supplement Figure 1. Anxiety degree and cognitive impairment in groups of *P.g* mice after gavage for 8weeks. (A, B) Total distance and velocity of mice moving in the Y-maze test. (C, D) Total distance and velocity of mice moving in the NORT. Two-group comparisons were performed using the unpaired *t*-test. Data are presented as the mean ± SEM, **P* < 0.05, ***P* < 0.01 *vs.* corresponding controls. Con, control; *P.g*, *Porphyromonas gingivalis*.

**Supplement Figure 2**

**
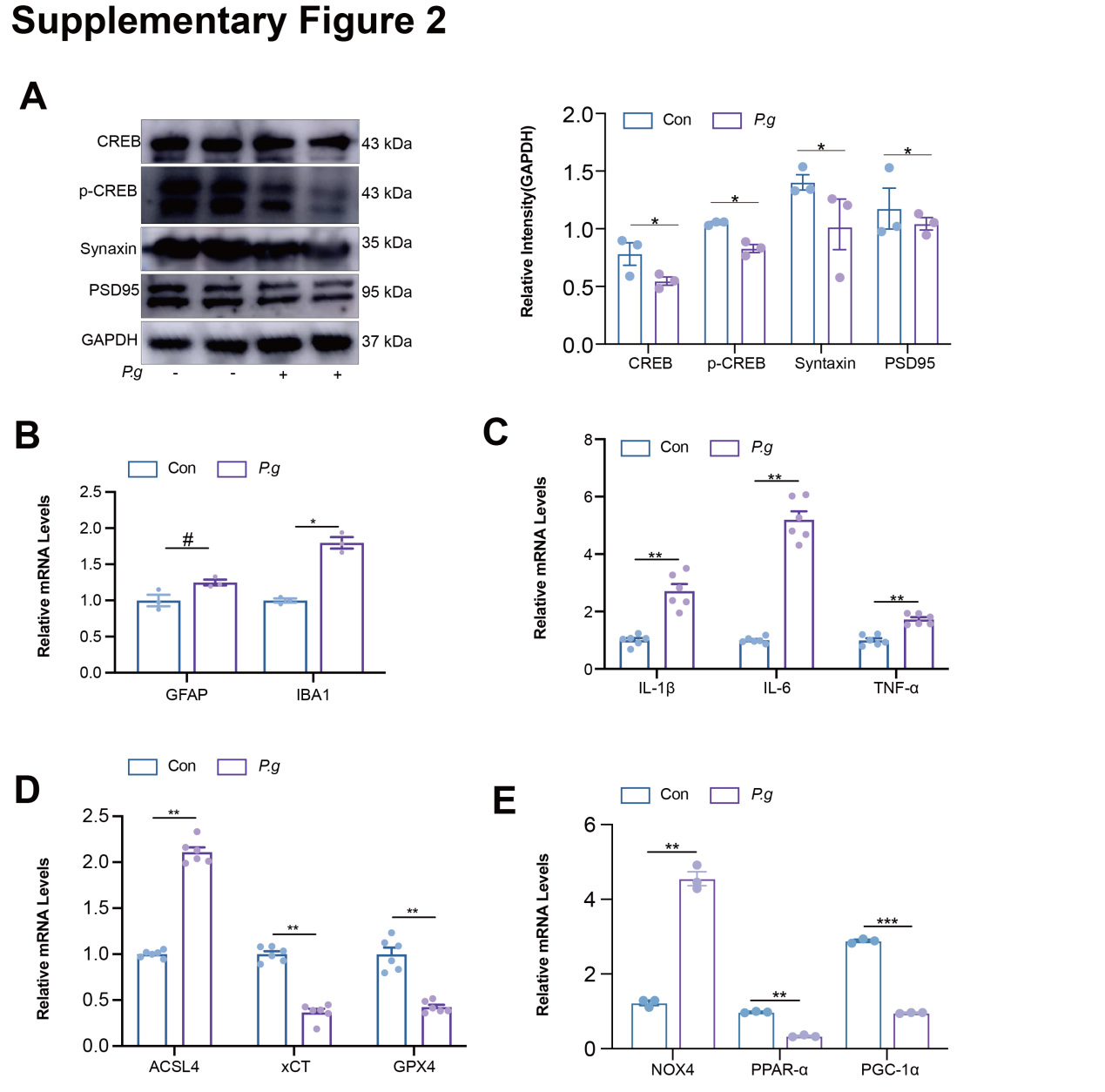
**

Supplement Figure 2. *P.g* promotes ferroptosis and neuroinflammation in mice. (A) Western blotting images and quantitative analyses of the expression levels of CREB, p-CREB, syntaxin, PSD-95. Two-group comparisons were performed using the unpaired *t*-test. (B-E) Relative mRNA expressions of immune cells, inflammation, ferroptosis, mitochondrial-related genes in brain tissues. Data are presented as the mean ± SEM, **P* < 0.05, ***P* < 0.01 *vs.* corresponding controls. Con, control; *P.g*, *Porphyromonas gingivalis*.

**Supplement Figure 3**

**
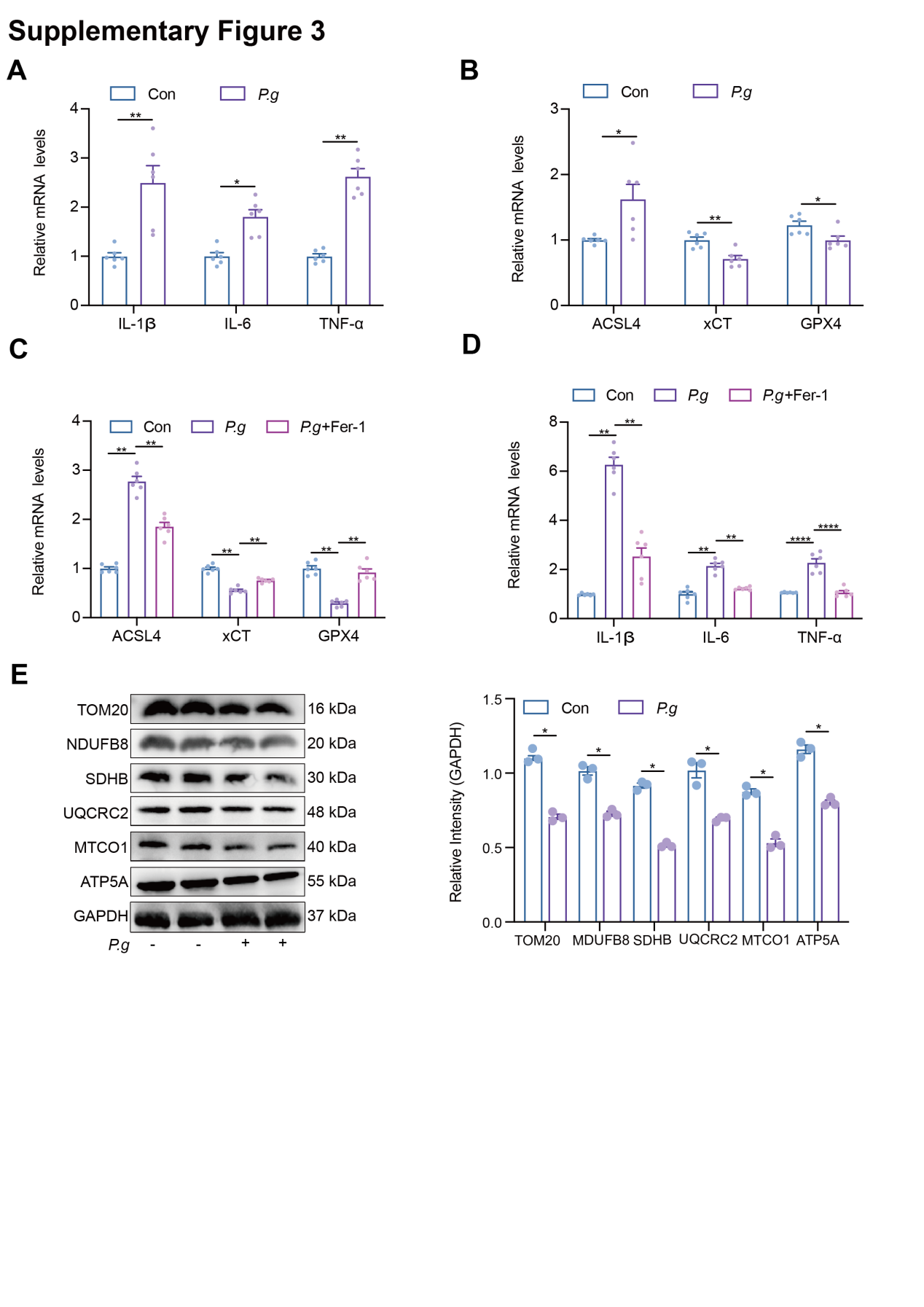
**

Supplement Figure 3. *P.g* promotes ferroptosis and neuroinflammation in HMC3. (A, B) Relative mRNA expressions of inflammation and ferroptosis in HMC3. (C, D) Relative mRNA expressions of inflammation and ferroptosis in Fer-1 treated HMC3. (E, F) Western blotting images and quantitative analyses of the expression levels of TOM20 and mitochondrial ETC protein levels. Two-group comparisons were performed using the unpaired *t*-test. Multi-group comparisons were performed using one-way ANOVA. Data are presented as the mean ± SEM, **P* < 0.05, ***P* < 0.01 *vs.* corresponding controls. Con, control; *P.g*, *Porphyromonas gingivalis*.

**Supplement Figure 4**

**
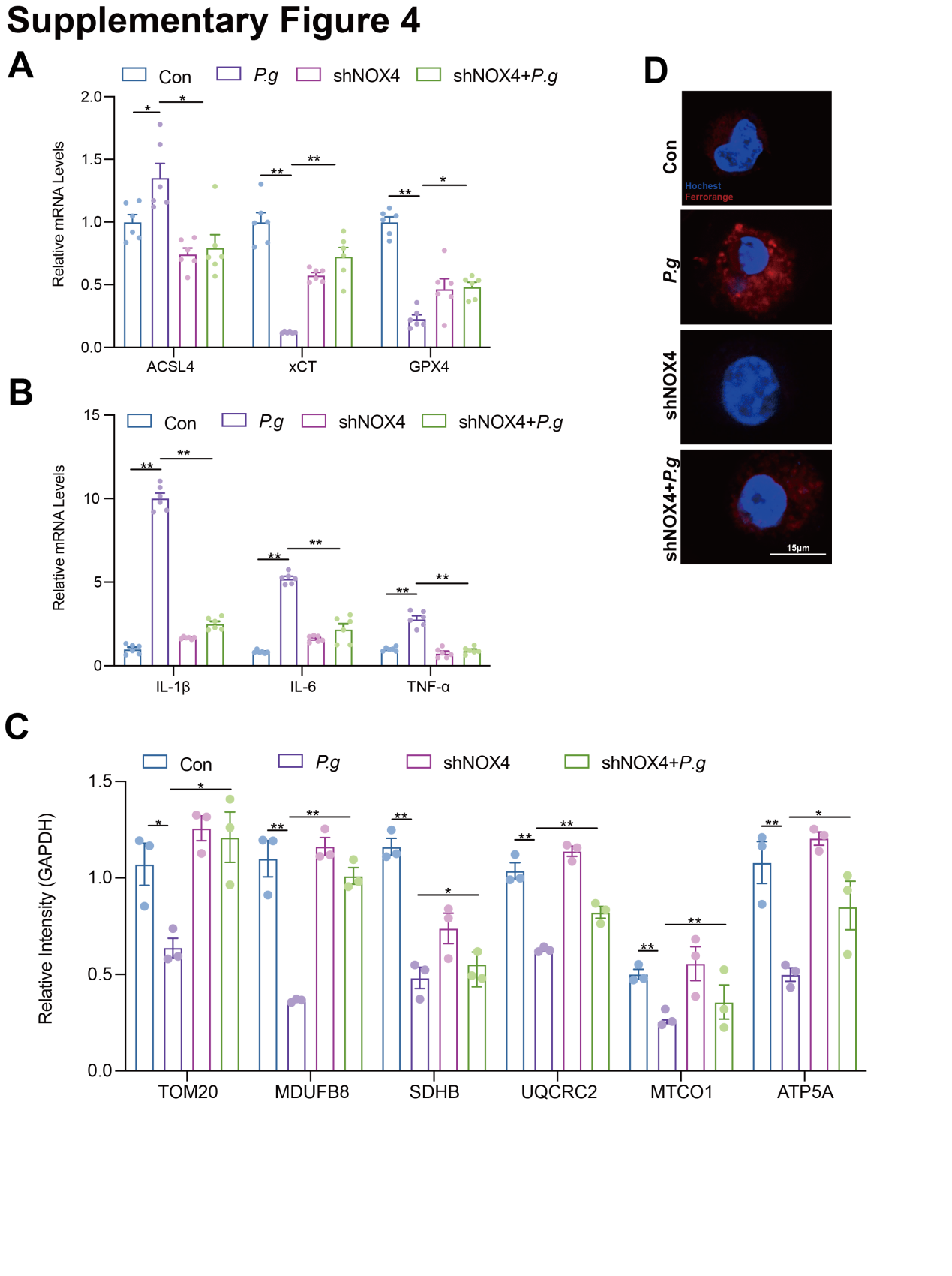
**

Supplement Figure 4. ShNOX4 prevents ferroptosis and inflammation in HMC3. (A, B) Relative mRNA expressions of inflammation and ferroptosis in HMC3. (C) Quantitative analyses of the expression proteins levels of TOM20 and mitochondrial ETC protein levels. (D) Staining of FerroOrange in microglia. Multi-group comparisons were performed using one-way ANOVA. Data are presented as the mean ± SEM, **P* < 0.05, ***P* < 0.01 *vs.* corresponding controls. Con, control; *P.g*, *Porphyromonas gingivalis*; shNOX4, NOX4 post-transfection; shNOX4+*P.g*, NOX4 post-transfection+*Porphyromonas gingivalis*.

**Supplement Figure 5**

**
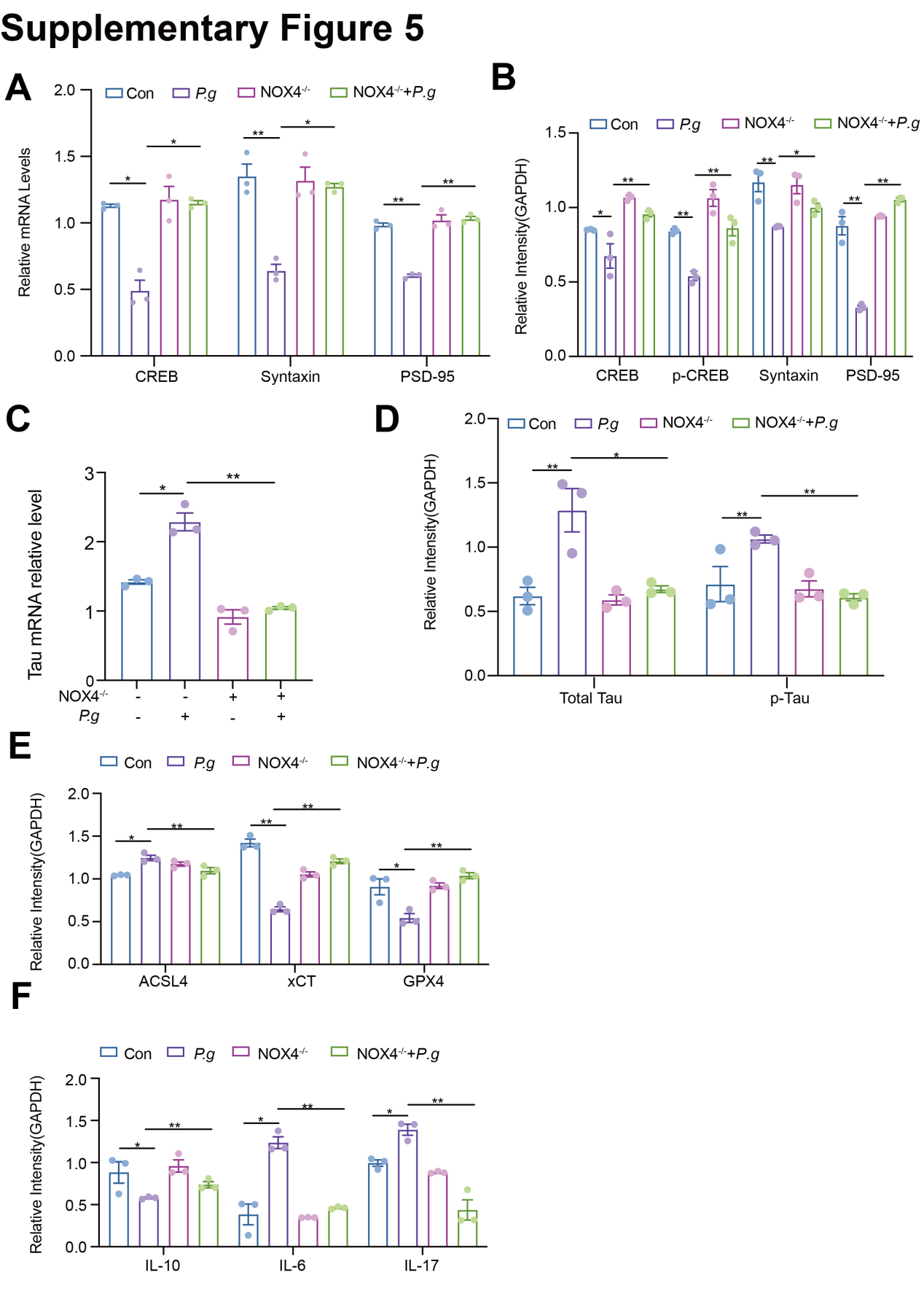
**

Supplement Figure 5. NOX4^-/-^ salvages the *P.g*-induced ferroptosis, neuroinflammation and pathologies. (A, C) Relative mRNA expressions of CREB, p-CREB, syntaxin, PSD-95 and Tau in brain tissues. (B, D-F) Quantitative analyses of the expression proteins levels of CREB, p-CREB, syntaxin, PSD-95, Tau, p-Tau, ferroptosis and inflammation.Multi-group comparisons were performed using one-way ANOVA. Data are presented as the mean ± SEM, **P* < 0.05, ***P* < 0.01 *vs.* corresponding controls. Con, control; *P.g*, *Porphyromonas gingivalis*; shNOX4, NOX4 post-transfection; shNOX4+*P.g*, NOX4 post-transfection+*Porphyromonas gingivalis*.

**Supplement Figure 6**

**
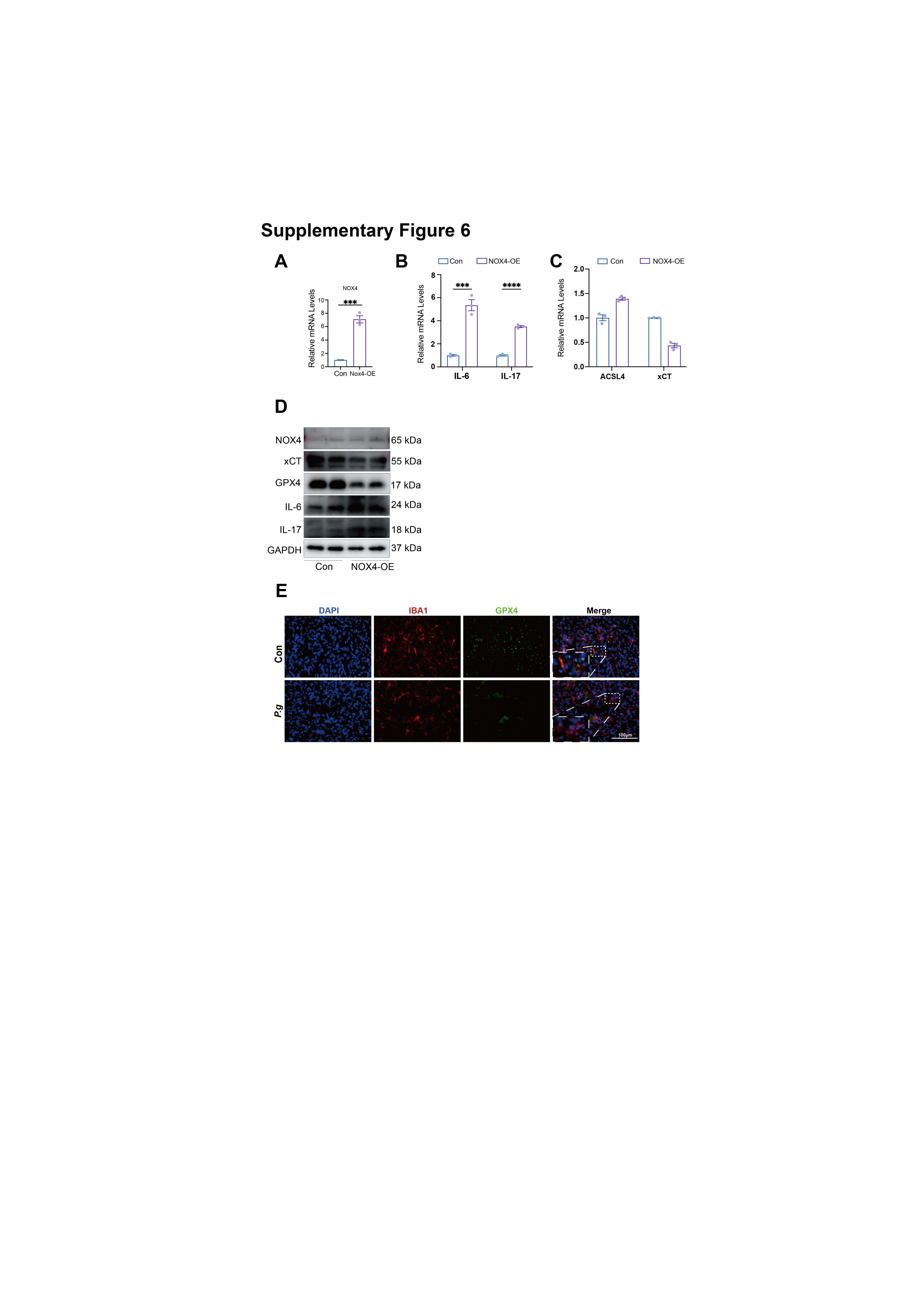
**

Supplement Figure 6. NOX4 overexpression promotes ferroptosis and neuroinflammation in HMC3. (A-C) Relative mRNA expressions of NOX4, inflammation and ferroptosis in HMC3. (D) Western blotting images of the expression of NOX4, inflammation and ferroptosis protein levels. (E) Co-staining of GPX4 and IBA1 in HMC3. Two-group comparisons were performed using the unpaired *t*-test. Multi-group comparisons were performed using one-way ANOVA. Data are presented as the mean ± SEM, **P* < 0.05, ***P* < 0.01 *vs.* corresponding controls. Con, control; NOX4-OE, HMC3 NOX4 overexpression.
