# Supplementary figures and images for: Porphyromonas gingivalis Promotes Neuroinflammation by Microglial Ferroptosis via NOX4/PPAR-α/PGC-1α Pathway
Source: Research (Wash D C). 2026 Apr 8;9:1163. doi: 10.34133/research.1163 (PMC13058221; doi:10.34133/research.1163)

# Supplementary Figure 1

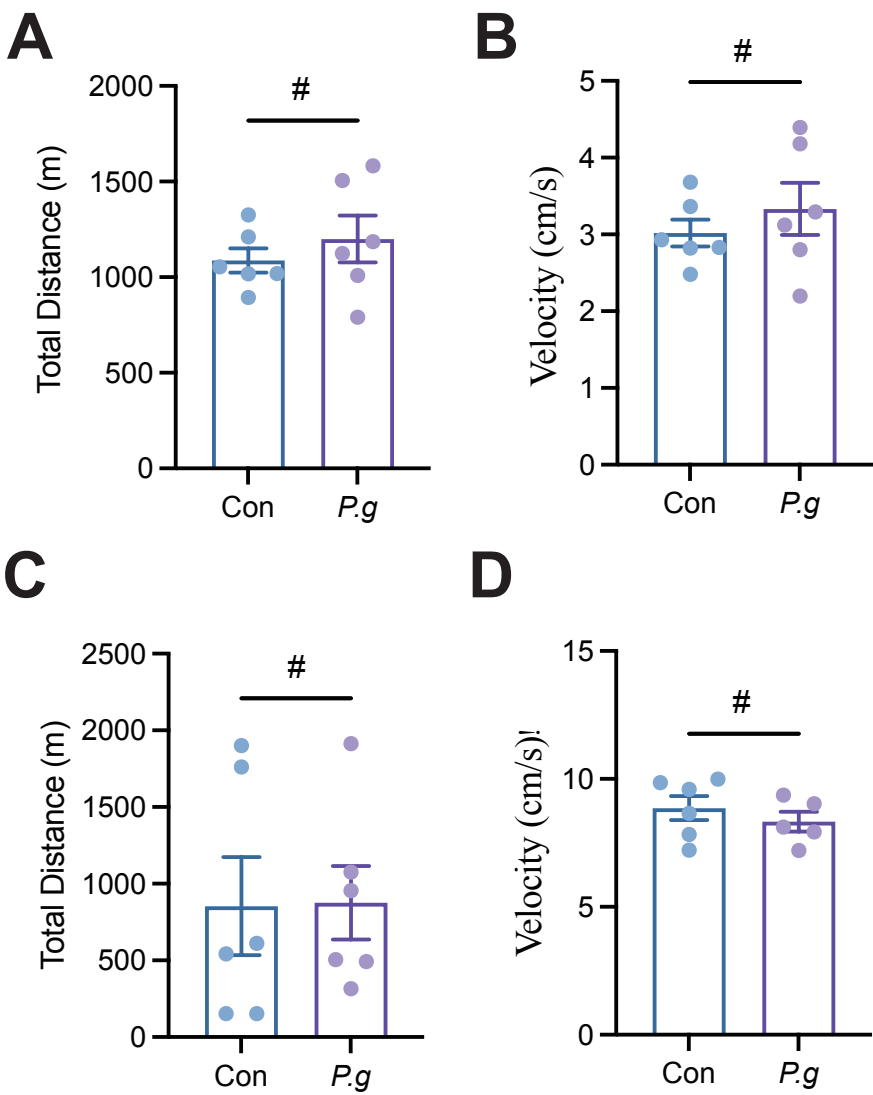

Supplement: Supplementary 1 — Figs. S1 to S6 [file research.1163.f1.zip › Sup 1.pdf]

# Supplementary Figure 2

**A**

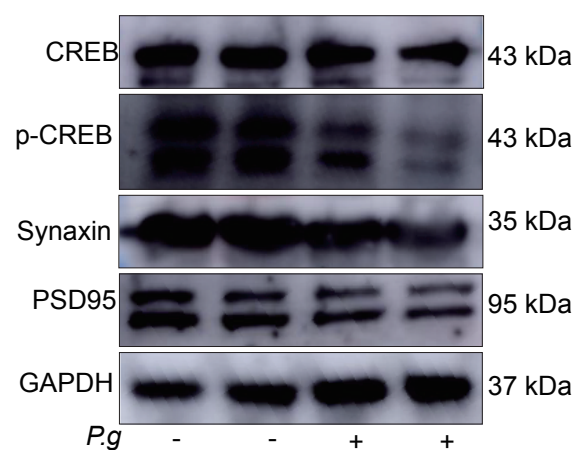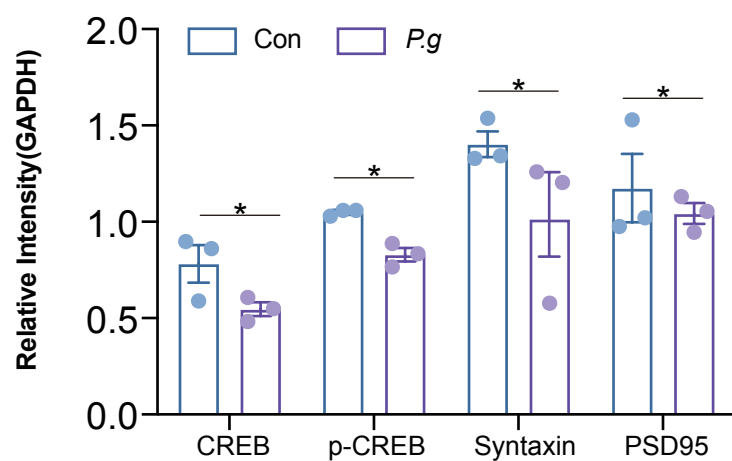

**B**

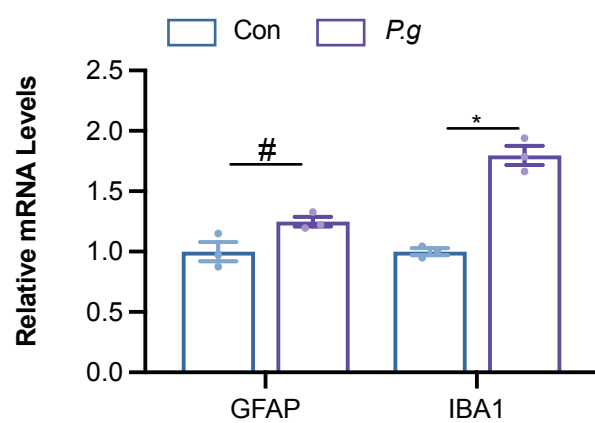

**C**

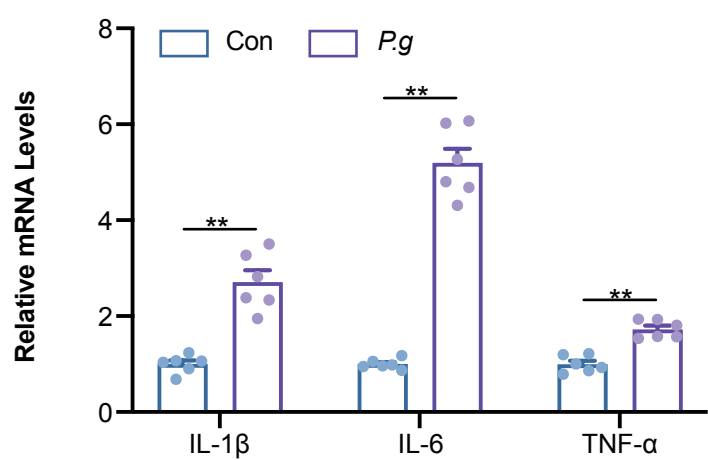

**D**

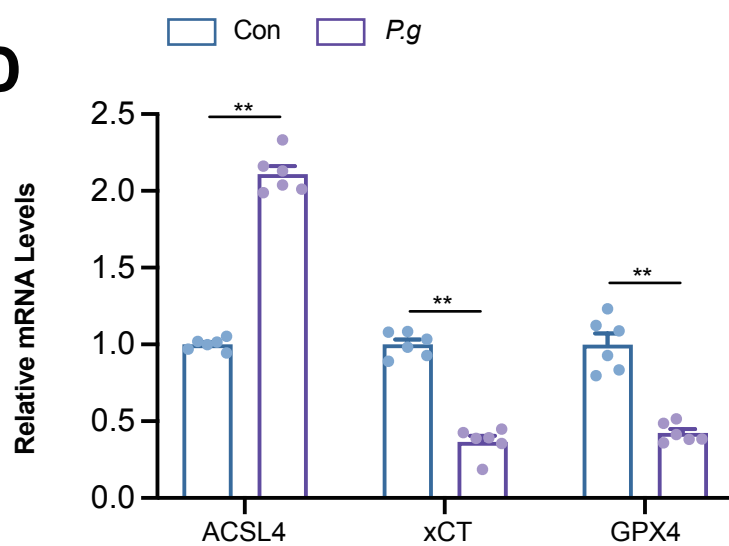

**E**

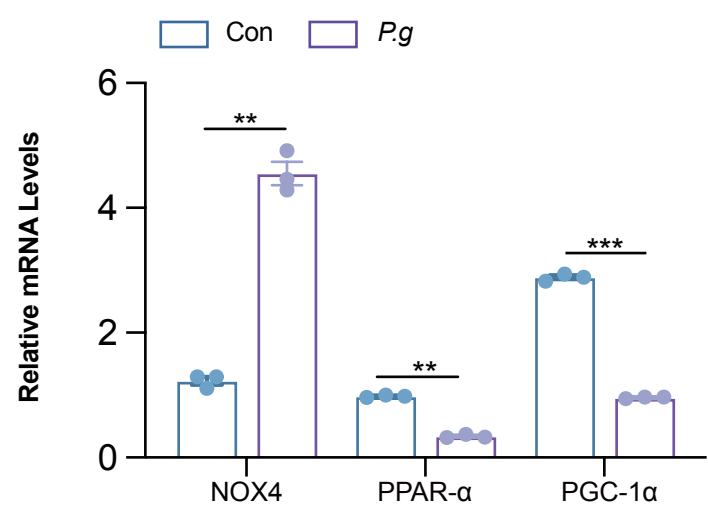

Supplement: Supplementary 1 — Figs. S1 to S6 [file research.1163.f1.zip › Sup 2.pdf]

Supplementary Figure 3

A

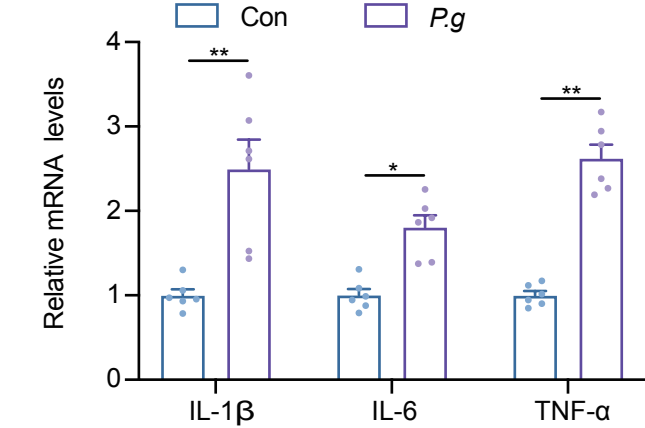

B

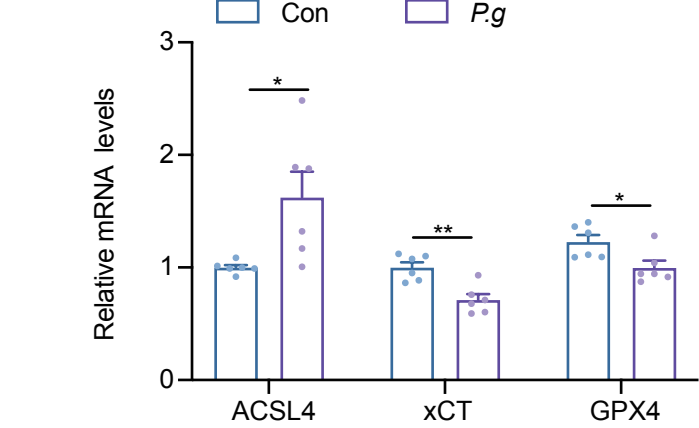

C

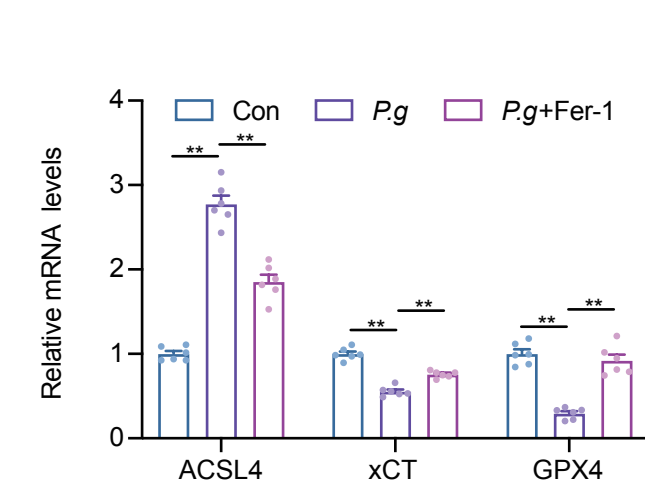

D

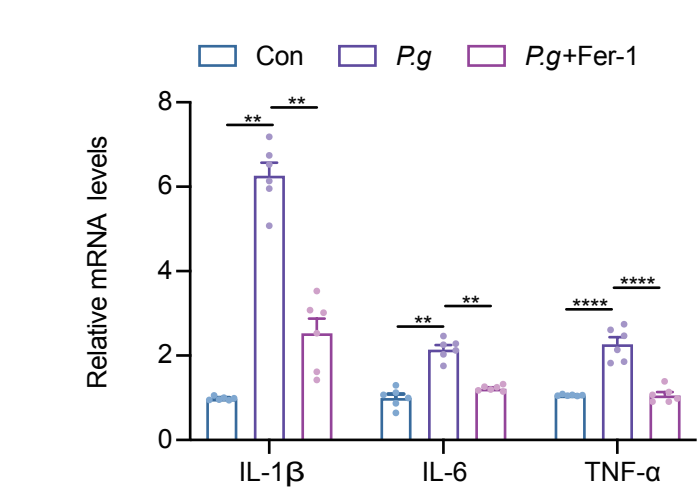

E

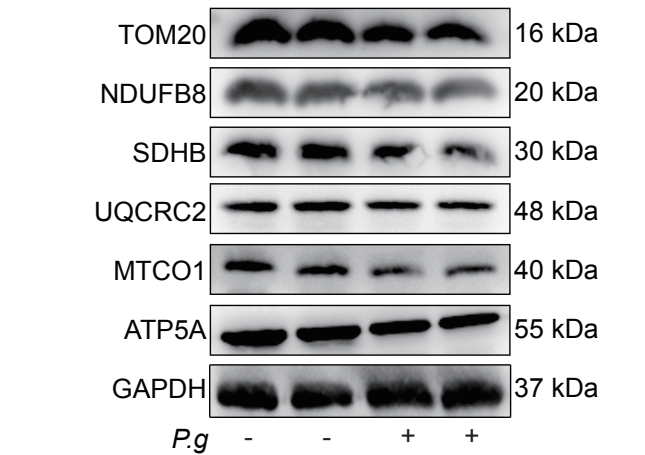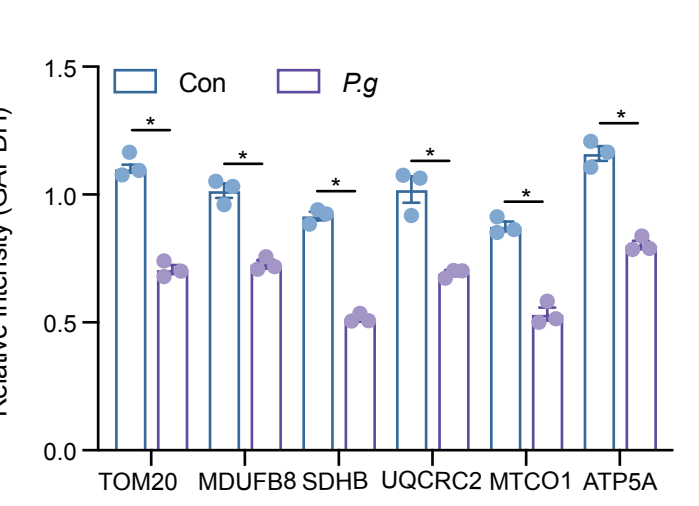

Supplement: Supplementary 1 — Figs. S1 to S6 [file research.1163.f1.zip › Sup 3.pdf]

# Supplementary Figure 4

**A**

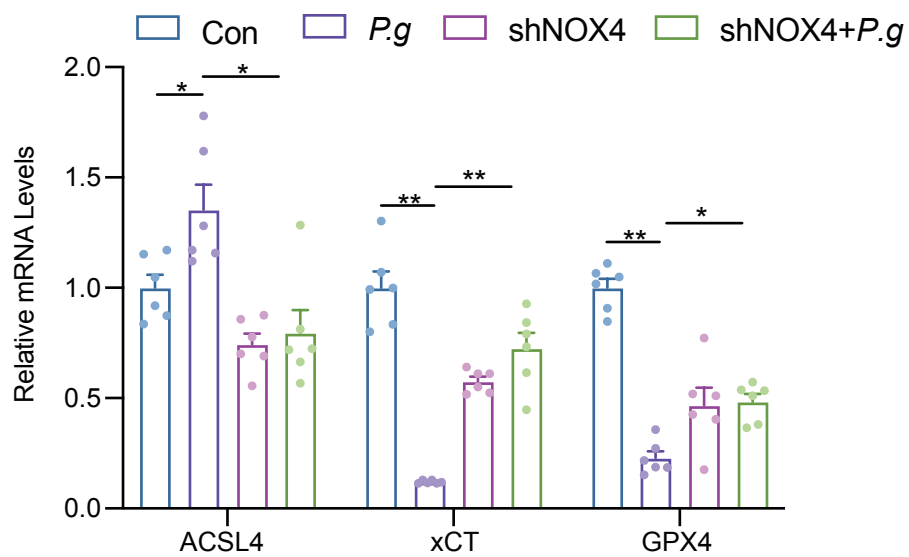

**B**

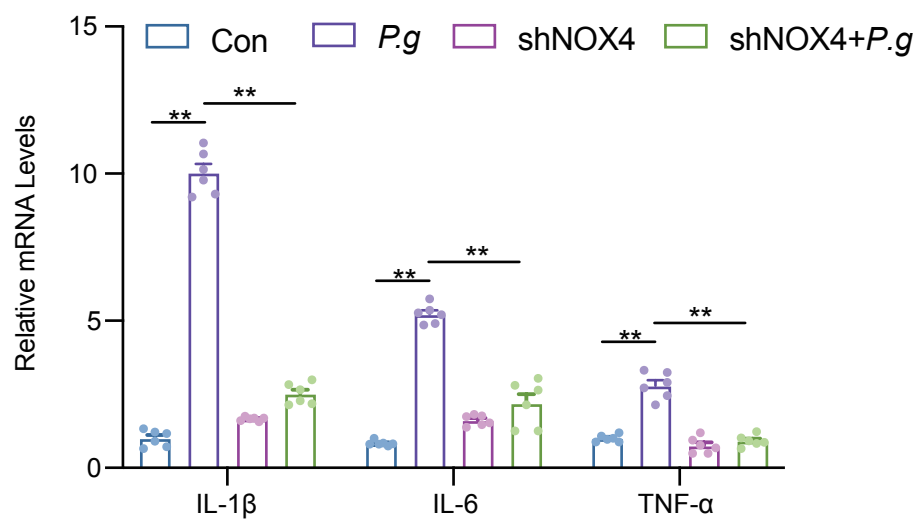

**C**

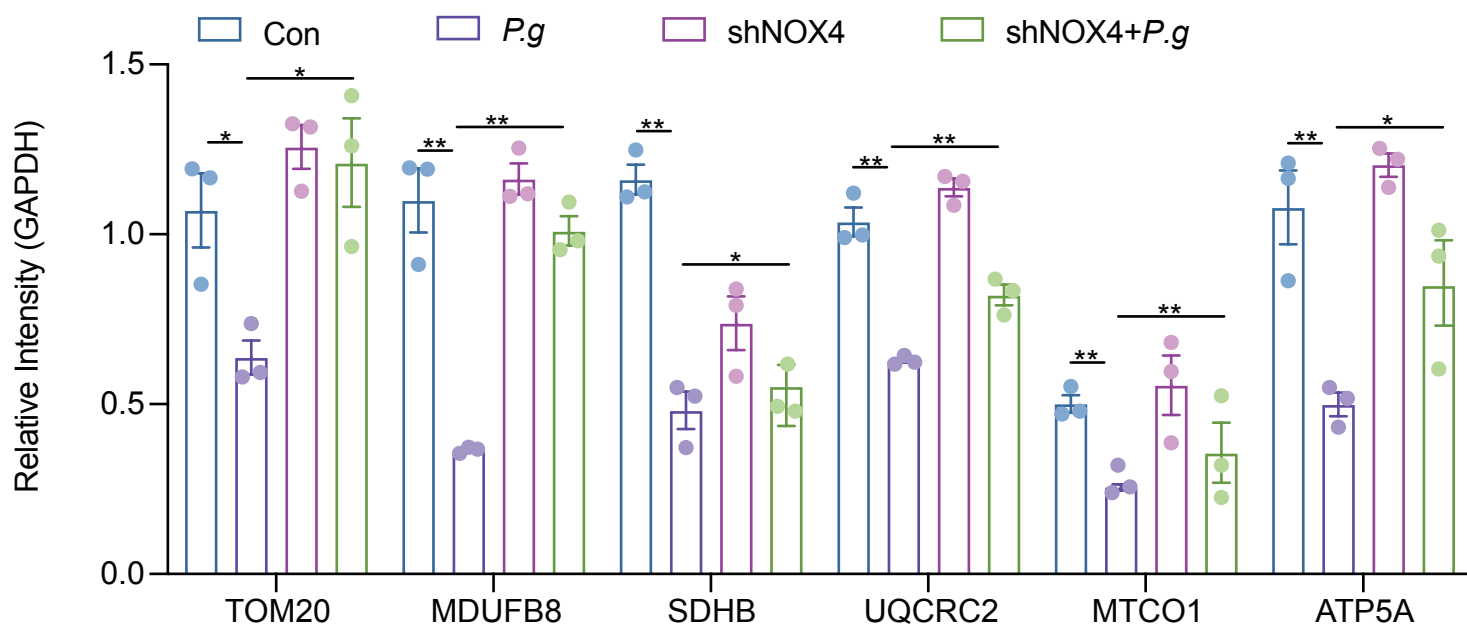

**D**

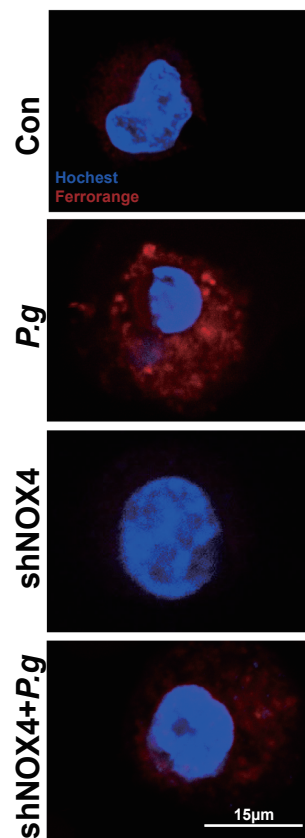

Supplement: Supplementary 1 — Figs. S1 to S6 [file research.1163.f1.zip › Sup 4.pdf]

# Supplementary Figure 5

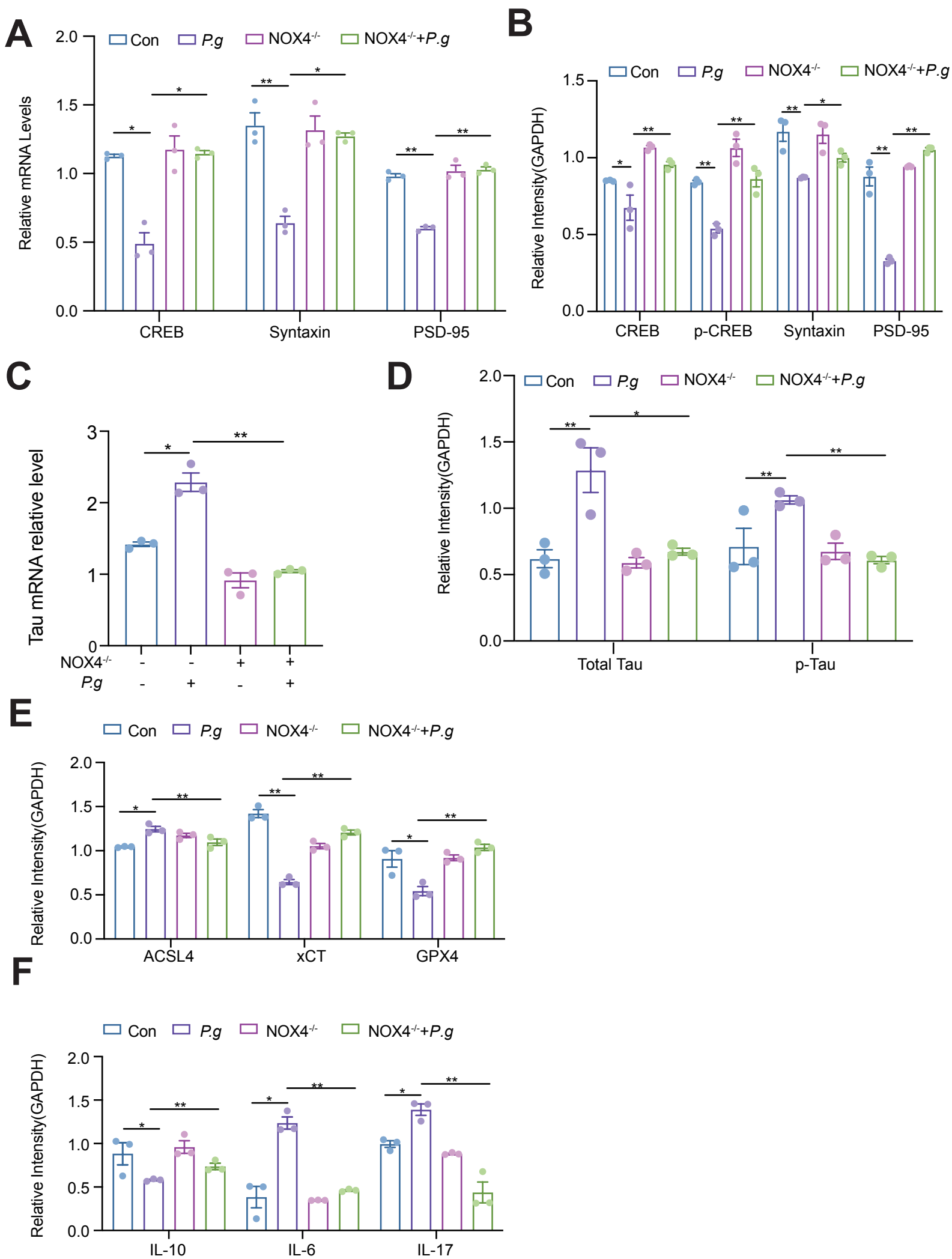

Supplement: Supplementary 1 — Figs. S1 to S6 [file research.1163.f1.zip › Sup 5.pdf]

# Supplementary Figure 6

**A**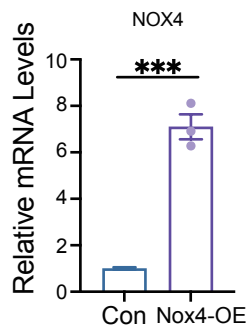**B**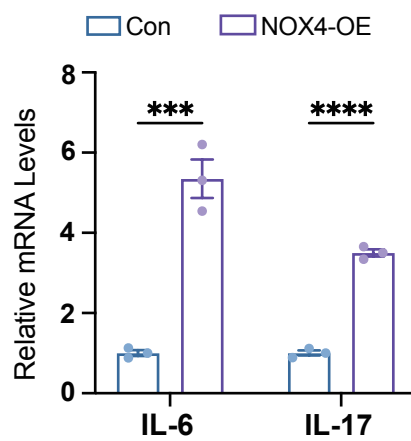**C**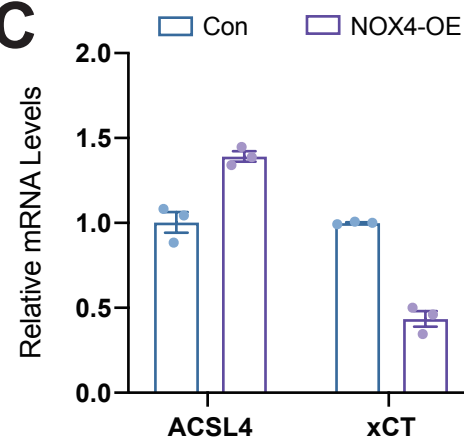**D**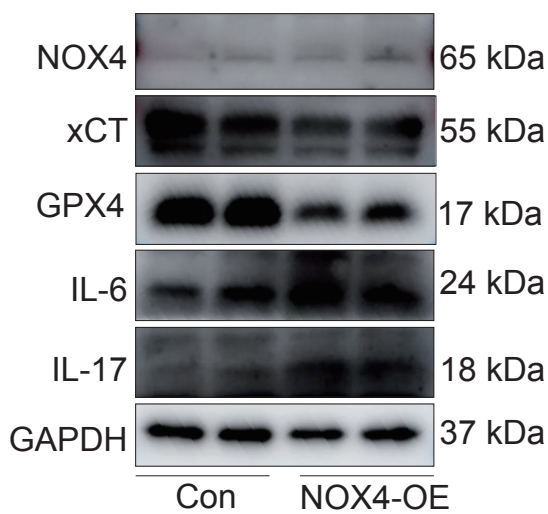**E**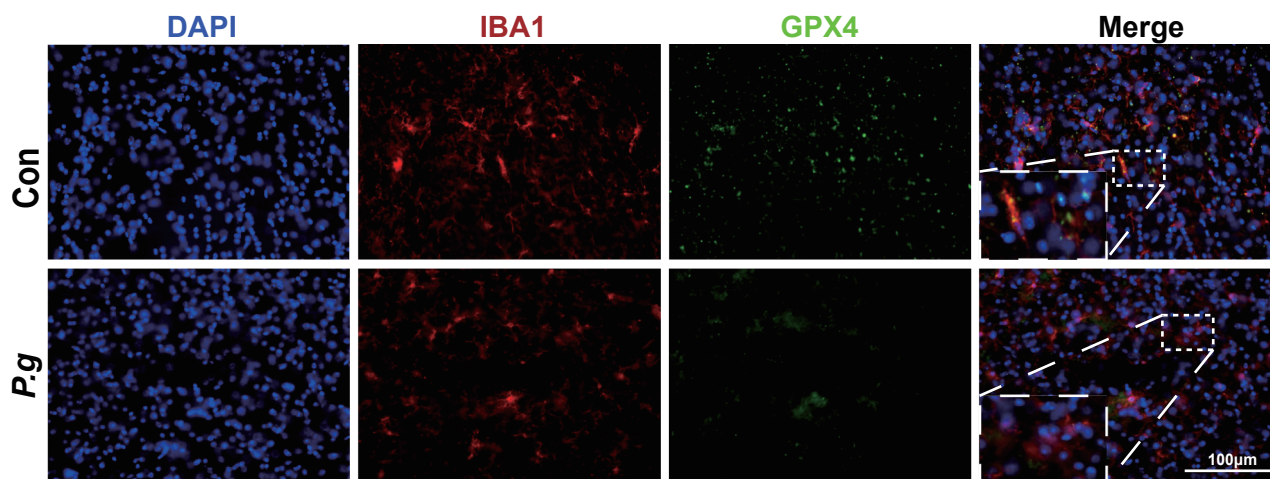

Supplement: Supplementary 1 — Figs. S1 to S6 [file research.1163.f1.zip › Sup6.pdf]
